# Cross-national Dataset on Psychological, Cognitive, and Contextual Factors of Entrepreneurial Behavior in Business Students

**DOI:** 10.12688/f1000research.168958.1

**Published:** 2025-09-18

**Authors:** Marisleidy Alba, Boris Cendales, Angélica Garzón Umerenkova, Juan Carlos Espinosa, Janitza Ariza Salazar

**Affiliations:** 1 Fundacion Universitaria Konrad Lorenz, Bogotá, Bogota, Colombia; 2 Universidad El Bosque, Bogotá, Bogota, Colombia; 3 Fundacion Universitaria Konrad Lorenz, Bogotá, Bogota, Colombia; 4 Universidad del Rosario, Bogotá, Bogota, Colombia; 5 Fundacion Universitaria Konrad Lorenz, Bogotá, Bogota, Colombia

**Keywords:** Adaptive curriculum, Cognitive load, Emotional competencies, Entrepreneurial action phases, Entrepreneurial intention, Financial knowledge, Entrepreneurial self-efficacy.

## Abstract

The study explores the factors that influence entrepreneurial intention and action among university students in five Latin American countries, integrating correlation analyses, multiple regressions, and visualizations through Raincloud Plots. Although numerous emotional, cognitive, and contextual profile variables were measured, this report presents only those that showed significant differences between countries or were identified as relevant predictors of entrepreneurial intention and action. Key variables such as entrepreneurial attitude, self-efficacy, entrepreneurial action in its discovery phase, university training, and empathy were identified as significant predictors of entrepreneurial intention. Self-awareness, self-efficacy, and entrepreneurial attitude explained the discovery phase. In contrast, financial knowledge, emotional well-being, flow at work, self-efficacy, and low cognitive load were determinants in the exploitation phase. The findings highlight the need for an adaptive curriculum that incorporates emotional competencies, training pathways differentiated by entrepreneurial stage, and support strategies that include mentoring, early diagnosis, and well-being management.

## Introduction

Entrepreneurship in university contexts has emerged as a key factor for economic development, social innovation, and improved employability. Universities are not only institutions for developing technical skills, but also incubators of entrepreneurial capacities that enable students to identify opportunities, manage resources, and develop impactful projects ( Fayolle & Gailly, 2015; Lackéus, 2020).

From a psychological and behavioral perspective, entrepreneurial behavior can be understood through the Theory of Planned Behavior ( Ajzen, 1991), which posits that entrepreneurial intention is determined by variables such as attitude toward behavior, perceived control (self-efficacy, locus of control), and perceived social norms. Complementarily, competency-based approaches ( Fernández-Pérez et al., 2019) emphasize the role of emotions, self-regulation, and prior experience in shaping entrepreneurial intention and action.

This study, conducted with students from business schools accredited by the Accreditation Council for Business Schools and Programs (ACBSP) in five Latin American countries (Mexico, Peru, Ecuador, Paraguay, and Colombia), analyzes how psychological variables (such as attitude, self-efficacy, emotional competence, emotional well-being), cognitive variables (financial knowledge, cognitive load, flow), and contextual variables (university training, risk perception) relate to entrepreneurial intention and action—both in the discovery and exploitation phases.

To explore these relationships, non-parametric statistical techniques were used, given the ordinal nature of the data and the lack of normality in several evaluated variables. The Kruskal-Wallis test was applied to identify significant differences between countries.

Spearman’s rank correlation (ρ) was also used, allowing the identification of highly interrelated variables—particularly within the emotional domain—revealing psychological profiles and influence patterns that are key to understanding the entrepreneurial phenomenon in the region. Potential predictors of entrepreneurial intention and action were examined using multiple linear regressions to determine the variables with the most significant explanatory power.

Visual techniques such as raincloud plots were incorporated to represent the distribution of the analyzed variables by country, integrating density diagrams, individual scores, and central tendency measures.

Overall, the findings of this report aim to provide a comprehensive and comparative perspective on the factors influencing university students’ entrepreneurial behavior in Latin America, to inform the design of more effective institutional strategies to foster entrepreneurship from a contextual, emotional, and cognitive standpoint.

## Method

### Participants

The sample for this study consisted of undergraduate students enrolled in business schools accredited by the ACBSP in four Latin American countries: Mexico (n = 170), Peru (n = 100), Ecuador (n = 147), Paraguay (n = 82), and Colombia (n = 42). A voluntary and deterministic sampling method was employed, with inclusion criteria requiring participants to be over 18 years of age and to have completed at least one entrepreneurship-related course at their educational institution. Data collection was conducted between May 2024 and May 2025 through coordinated recruitment with program directors, including invitations sent via institutional email, announcements in virtual classrooms, and direct communication in scheduled lectures.

A total of 541 valid responses were obtained. The study employed a correlational, cross-sectional, and non-experimental design. The self-administered online survey was delivered through a secure platform and required approximately 15–20 minutes to complete.

Written informed consent was obtained from all participants before data collection. For the online survey, consent was documented through a mandatory digital acceptance form displayed before the questionnaire, in which participants confirmed their willingness to take part in the study and acknowledged the confidentiality terms. All participants were over 18 years of age; therefore, no parental consent or minor assent procedures were necessary. Participation was entirely voluntary, and the research instrument did not cause any discomfort, physical, or psychological harm. The study was conducted fairly and equitably, ensuring gender balance in the sample. Data confidentiality and anonymity were guaranteed by Decree 1377 of 2013, Law 1581 of 2012, Andean Decision 351 of 1993, and the institutional policy on personal data management.

### Measures

*Measurement instruments*

A set of validated questionnaires was administered, organized into four blocks: psychological, cognitive, contextual, and entrepreneurial. All scales used a 5-point Likert format (1 = Strongly disagree, 5 = Strongly agree), with scores calculated as the mean of the items. The Spanish versions were reviewed through a translation–back translation process and validated by experts. Average Variance Extracted (AVE) and reliability (Cronbach’s α and McDonald’s ω) for the present sample are reported below.

1. Psychological variables
  • Entrepreneurial Attitude (PromEA): 8 items ( Song et al., 2021), e.g., “I want to be an agent of change in society” (AVE = 0.665, α = 0.920, ω = 0.929).
  • Entrepreneurial Self-Efficacy (PromSE): 5 items ( Fernández-Pérez et al., 2019), e.g., “I feel capable of recognizing opportunities for the development of new products and/or services” (AVE = 0.850, α = 0.948, ω = 0.950).
  • Emotional Competencies (EC): three dimensions: self-awareness (3 items, AVE = 0.859, α = 0.928, ω = 0.906), empathy (5 items, AVE = 0.849, α = 0.947, ω = 0.951), social skills (5 items, AVE = 0.812, α = 0.938, ω = 0.936).
  • Emotional Well-being (PromEW): SWLS, five items ( Diener et al., 1985), e.g., “In most ways my life is close to my ideal” (AVE = 0.750, α = 0.920, ω = 0.924).
2. Cognitive variables
  • Financial Knowledge (FK): 8 items ( Chen & Volpe, 1998), e.g., “I am knowledgeable about personal financial planning” (AVE = 0.718, α = 0.933, ω = 0.949).
  • Cognitive Load (PromCL): 8 items ( Leppink et al., 2013) (AVE = 0.588, α = 0.582, ω = 0.142).
  • Flow (PromFL): 6 items, FMQ ( Wilson & Moneta, 2016), e.g., “My mind does not wander. I am totally involved in what I am doing and I am not thinking of anything else” (AVE = 0.603, α = 0.868, ω = 0.902).
3. Contextual variables
  • Perception of the University Environment (PromU): 6 items adapted from Franke & Lüthje (2004), e.g., “University courses … prepare you well to work on your own” (AVE = 0.742, α = 0.956).
  • Risk Propensity (PromR): 9 items ( Yurtkoru et al., 2014), e.g., “If I am afraid of something, I will try to overcome my fears” (α = 0.610, ω = 0.691).
4. Entrepreneurial variables
  • Entrepreneurial Intention (PromEI): 5 items (Liñán & Chen, 2009), e.g., “My professional goal is to become an entrepreneur” (AVE = 0.893, α = 0.960, ω = 0.966).
  • Entrepreneurial Action: 16 items ( Botha & Pietersen, 2020), two phases:
    ○ Discovery (5 items, AVE = 0.684, α = 0.884, ω = 0.891)
    ○ Exploitation (11 items, AVE = 0.800, α = 0.965, ω = 0.993).

### Statistical analysis

Data collection was conducted between May 2024 and May 2025 through recruitment strategies coordinated with program directors from accredited business schools, to ensure diversity in gender, age, and country representation, thereby reducing potential sampling bias.

Measures to minimize common method bias included:

(a) ensuring anonymity,
(b) informing participants of the absence of right or wrong answers, and
(c) randomizing the order of items within each block.

Confidentiality and anonymity were maintained to reduce social desirability bias.

The statistical analysis proceeded in several stages:

1. Exploratory Factor Analysis (EFA):
  • Conducted separately for each instrument to evaluate its internal structure.
  • Extraction method: minimum residuals with Promax oblique rotation, appropriate for potentially correlated factors.
  • Input: polychoric correlation matrix (due to the ordinal nature of the data).
  • Retention criteria: eigenvalues > 1, scree plot inspection, and parallel analysis.
  • Items with factor loadings < 0.40 or cross-loadings > 0.30 were flagged for potential removal.
2. Scale scoring:
  • Composite scores for each variable were calculated as the arithmetic mean of the retained items per participant.
  • Negatively worded items were reverse-coded before computing the mean score.
3. Normality assessment:
  • The Shapiro–Wilk test was applied within each country group.
  • Skewness and kurtosis statistics, as well as histograms, were inspected.
  • Given violations of normality, the ordinal nature of Likert-type items ( Norman, 2010), and the presence of outliers, non-parametric methods were selected.
4. Group comparisons:
  • The Kruskal–Wallis H test was used to compare medians across countries.
  • Significant results were followed by Dunn’s post-hoc tests with Bonferroni and Holm corrections for multiple comparisons.
5. Visualization:
  • Raincloud plots were created in JASP, combining:
    (a) jittered raw data points representing individual participants’ responses,
    (b) half-violin density curves illustrating the distribution and dispersion of responses, and
    (c) boxplots indicating the median and interquartile range.
6. Associations and predictors:
  • Spearman’s rank-order correlation (ρ) was used to assess bivariate associations between variables.
  • Multiple linear regression models were conducted in Jamovi to identify predictors of entrepreneurial intention and action (discovery and exploitation phases), using the Enter method and reporting standardized β coefficients, p-values, and adjusted R ^2^.

All analyses were conducted in JASP (version 0.19.3.0) and Jamovi (version 2.6.44).

## Results and Discussion

### Risk

**Figure 1.**
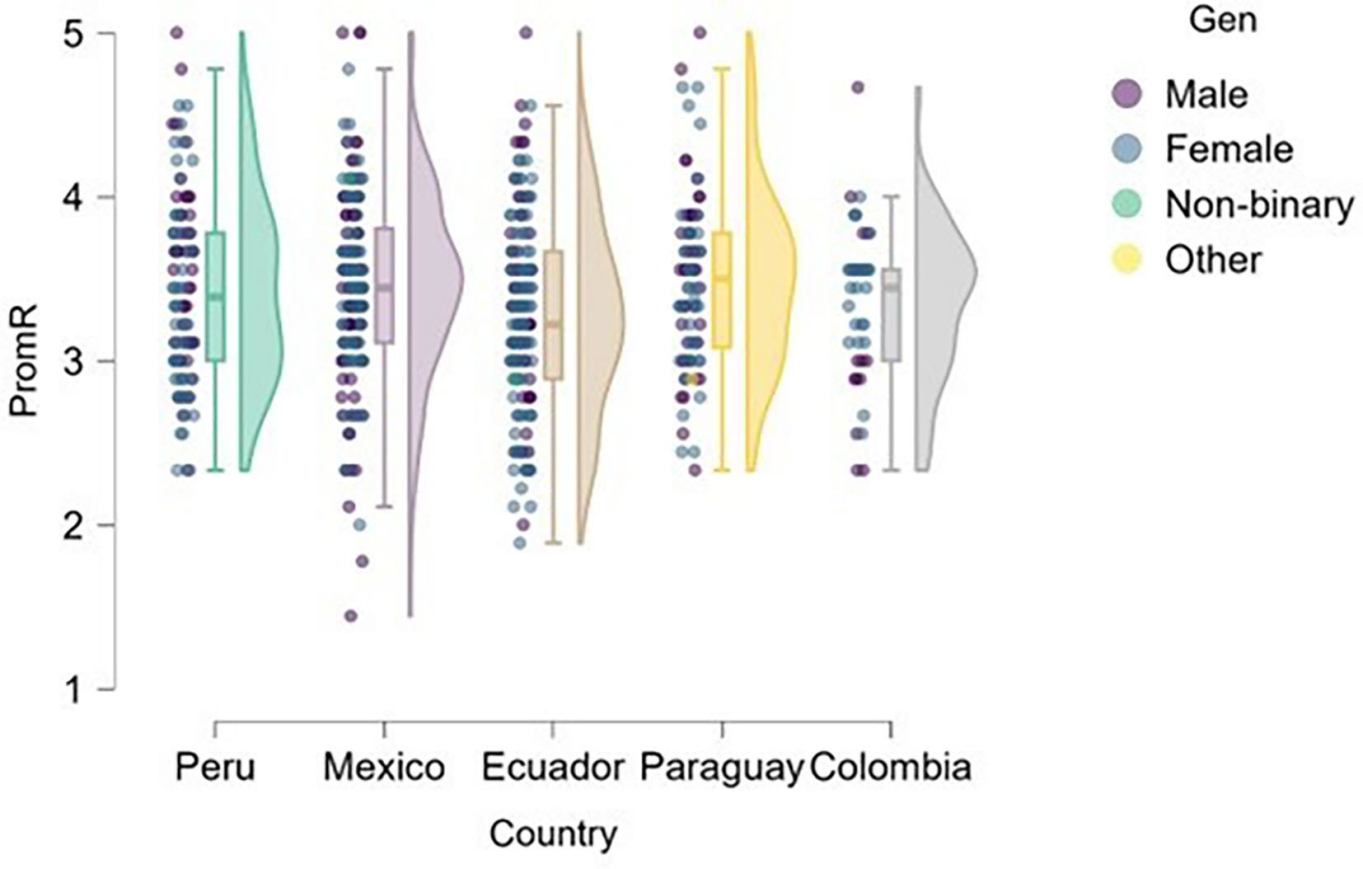
Risk perception by gender and country. Raincloud plots showing the distribution of risk perception scores among male and female undergraduate business students in Mexico (n = 170), Peru (n = 100), Ecuador (n = 147), Paraguay (n = 82), and Colombia (n = 42). Plots combine half-violin density curves, boxplots (median, interquartile range), and jittered raw data points. Scores measured on a 5-point Likert scale (1 = strongly disagree, 5 = strongly agree).

Figure 1 presents the distribution of risk perception by gender and country, revealing noteworthy patterns that may be interpreted through a gender perspective. This raises the question of whether significant differences exist in risk-taking propensity between male and female students across the university contexts studied, and how these differences manifest in each country.

Differences in risk perception by gender and country were analyzed. Although no statistically significant differences were found in most cases (p > .05), group median values showed a non-significant numerical trend toward slightly lower scores among women in countries such as Mexico, Ecuador, and Colombia (medians between 3.0 and 3.5 on a 5-point scale), which may suggest lower risk-taking propensity in these contexts.

This result is consistent with previous findings that have documented a lower inclination toward risk among women, attributed to factors such as differential socialization, gender role expectations, and the perception of uncertainty ( Croson & Gneezy, 2009; Byrnes et al., 1999). While this study did not identify statistically significant differences, these patterns may be considered indicative and valuable as a foundation for future research on gender differences in entrepreneurial behavior.

### Entrepreneurial intention

**Figure 2.**
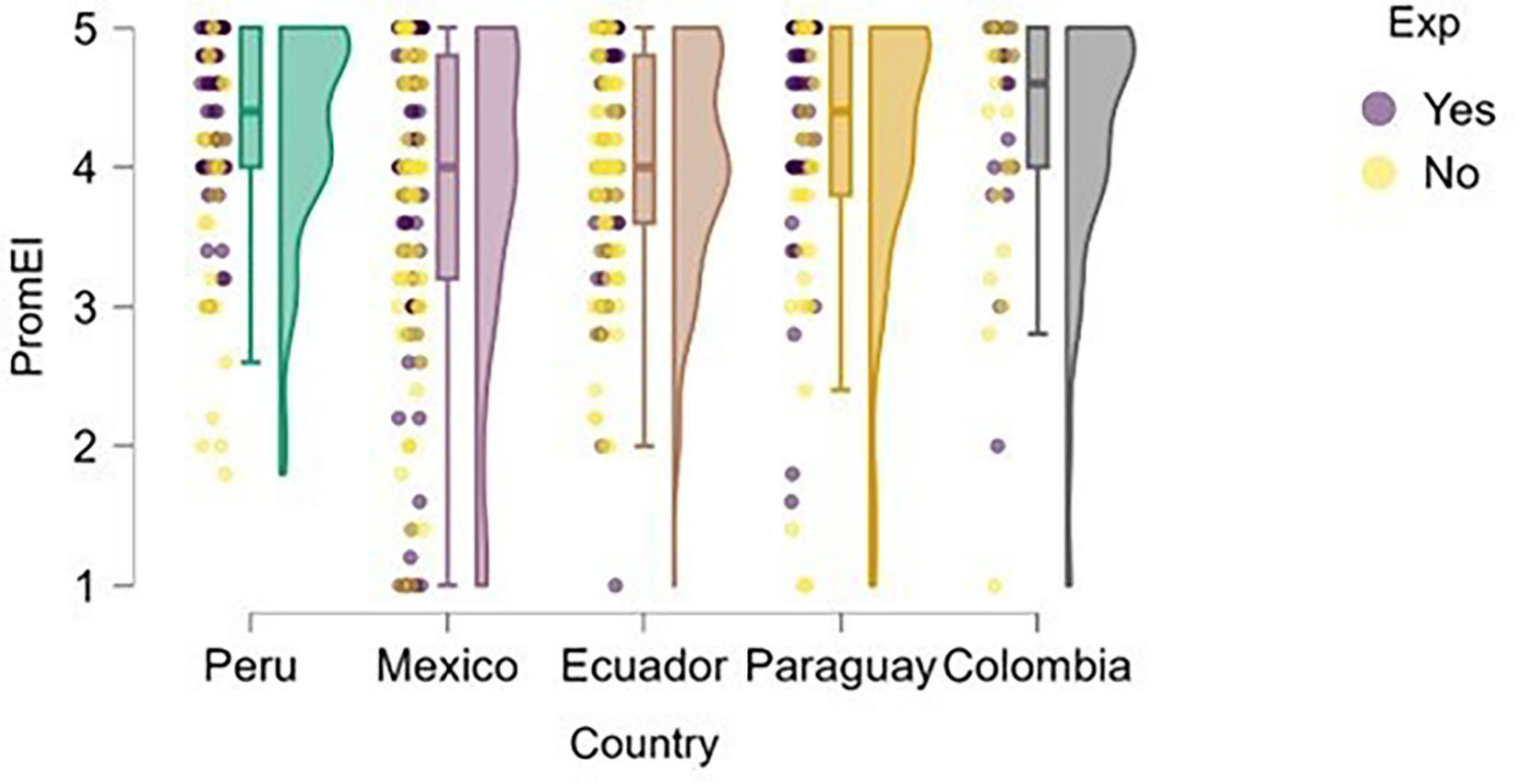
Entrepreneurial intention by country. Raincloud plots showing the distribution of entrepreneurial intention across the five countries. Colors indicate countries; plots combine density, boxplots, and raw points. Scores on a 5-point Likert scale.

On the other hand, Figure 2 illustrates the distribution of entrepreneurial intention across the countries studied. The results suggest that having prior entrepreneurial experience does not guarantee a higher level of entrepreneurial intention. This may indicate that experience alone does not significantly differentiate the development of intention within this sample. These findings support studies such as that of Kautonen et al. (2015), which argue that entrepreneurial intention is not solely dependent on past behavior, but instead emerges from a complex set of personal beliefs, attitudes, and subjective norms, as proposed by the Theory of Planned Behavior ( Ajzen, 1991).

Peru and Paraguay stood out as the countries with the highest levels of entrepreneurial intention among students, which may be attributed to sociocultural and institutional factors that foster proactive attitudes toward entrepreneurship. In contrast, students from Mexico and Ecuador showed a more dispersed distribution with a higher presence of low scores, suggesting a weaker consolidation of entrepreneurial intention in these contexts. Colombia, meanwhile, exhibited a high and homogeneous median, although without the extreme values observed in Peru. These differences may be related to the national entrepreneurial climate, perceived opportunities, or the type of training received.

### Entrepreneurial attitude

**Figure 3.**
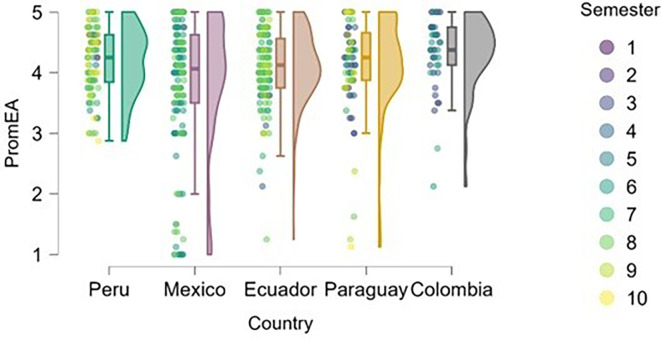
Entrepreneurial attitude by academic semester and country. Raincloud plots showing entrepreneurial attitude levels in relation to academic progress. Data grouped by country. Scores on a 5-point Likert scale.

Figure 3 reflects the trend that cumulative exposure to content, practical experiences, and entrepreneurial learning environments positively impacts entrepreneurial attitude—students in higher semesters reported greater levels of entrepreneurial attitude. This finding is consistent with the argument made by Lackéus (2020), who emphasizes that the development of entrepreneurial attitudes is an experiential process, intensified through real-world projects, mentorship, and engagement with the entrepreneurial ecosystem.

Students from Peru and Paraguay exhibited the highest and most homogeneous levels of entrepreneurial attitude. This suggests that the educational or cultural context may be actively fostering favorable dispositions toward entrepreneurship. In contrast, Mexico and Ecuador showed greater dispersion in responses and a higher proportion of students with low or moderate levels of entrepreneurial attitude, particularly among those in the early semesters. Colombia, on the other hand, displayed high levels of entrepreneurial attitude, though with lower density, indicating a more heterogeneous presence of this trait within the sample. According to Franke and Lüthje (2004), the university environment and perceptions of institutional support can significantly influence the development of entrepreneurial attitudes, which may help explain the observed differences across countries.

### University support

**Figure 4.**
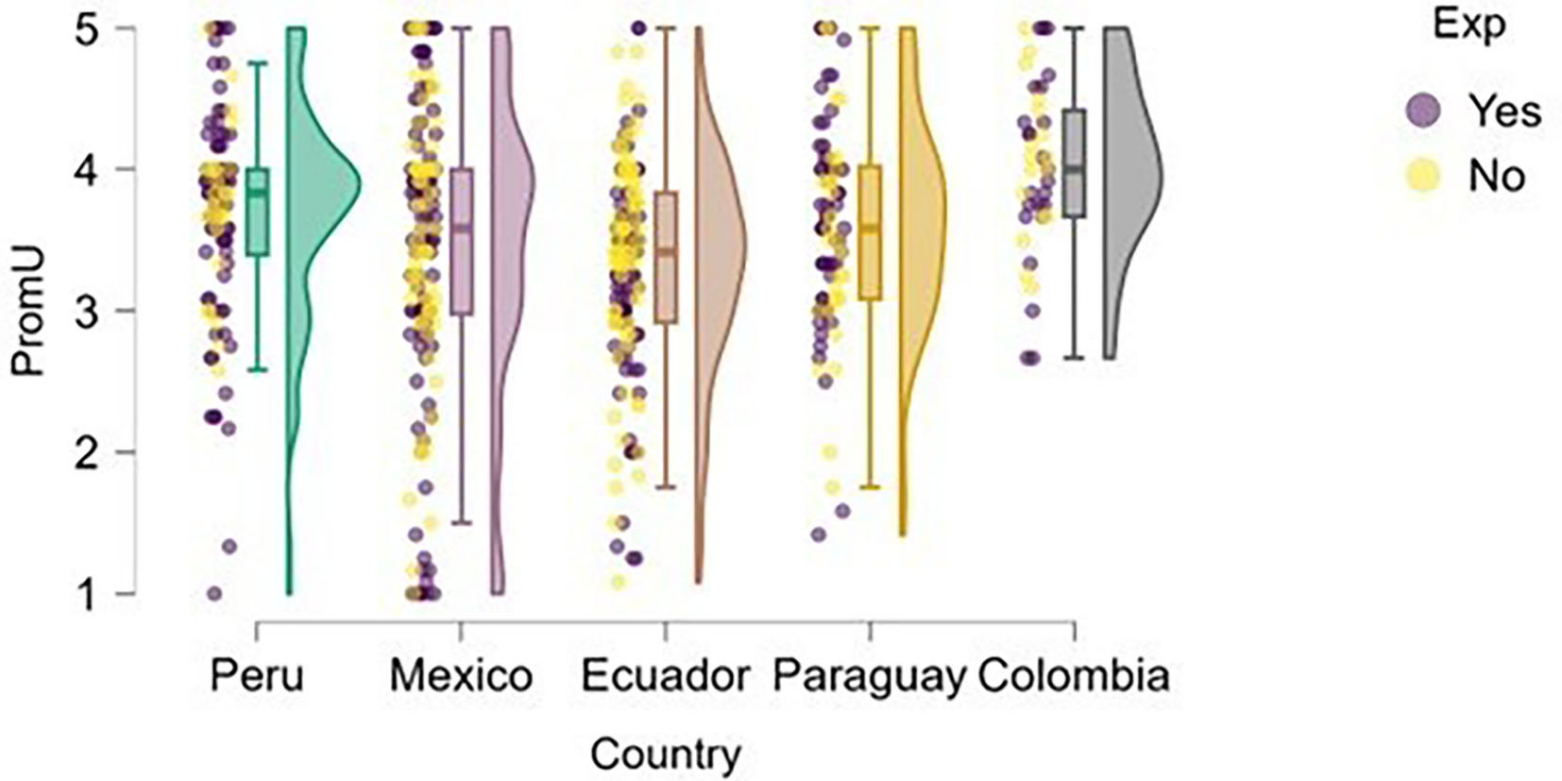
Perceived university support by entrepreneurial experience. Raincloud plots comparing perceived entrepreneurship education between students with and without prior entrepreneurial experience. Grouped by country; scores on a 5-point Likert scale.

Perception of university support was examined through the lens of training. Figure 4 illustrates how students’ perceptions of university-based entrepreneurship education vary according to their prior entrepreneurial experience. Overall, in countries such as Peru and Colombia, both students with and without entrepreneurial experience rated the training received positively, with high medians and low dispersion, suggesting the presence of well-established educational programs.

In contrast, in Mexico, Ecuador, and Paraguay, although median scores remain within moderate ranges, the dispersion is greater, and no substantial differences are observed between students with and without entrepreneurial experience. This may indicate that, while entrepreneurship education is present, it does not consistently translate into a differentiated perception of its usefulness or applicability. These results suggest that entrepreneurial experience alone does not determine a more favorable evaluation of university training, which may be linked to factors such as curriculum quality, the practical orientation of the program, or its alignment with the real needs of the local entrepreneurial ecosystem.

### Self-efficacy

**Figure 5.**
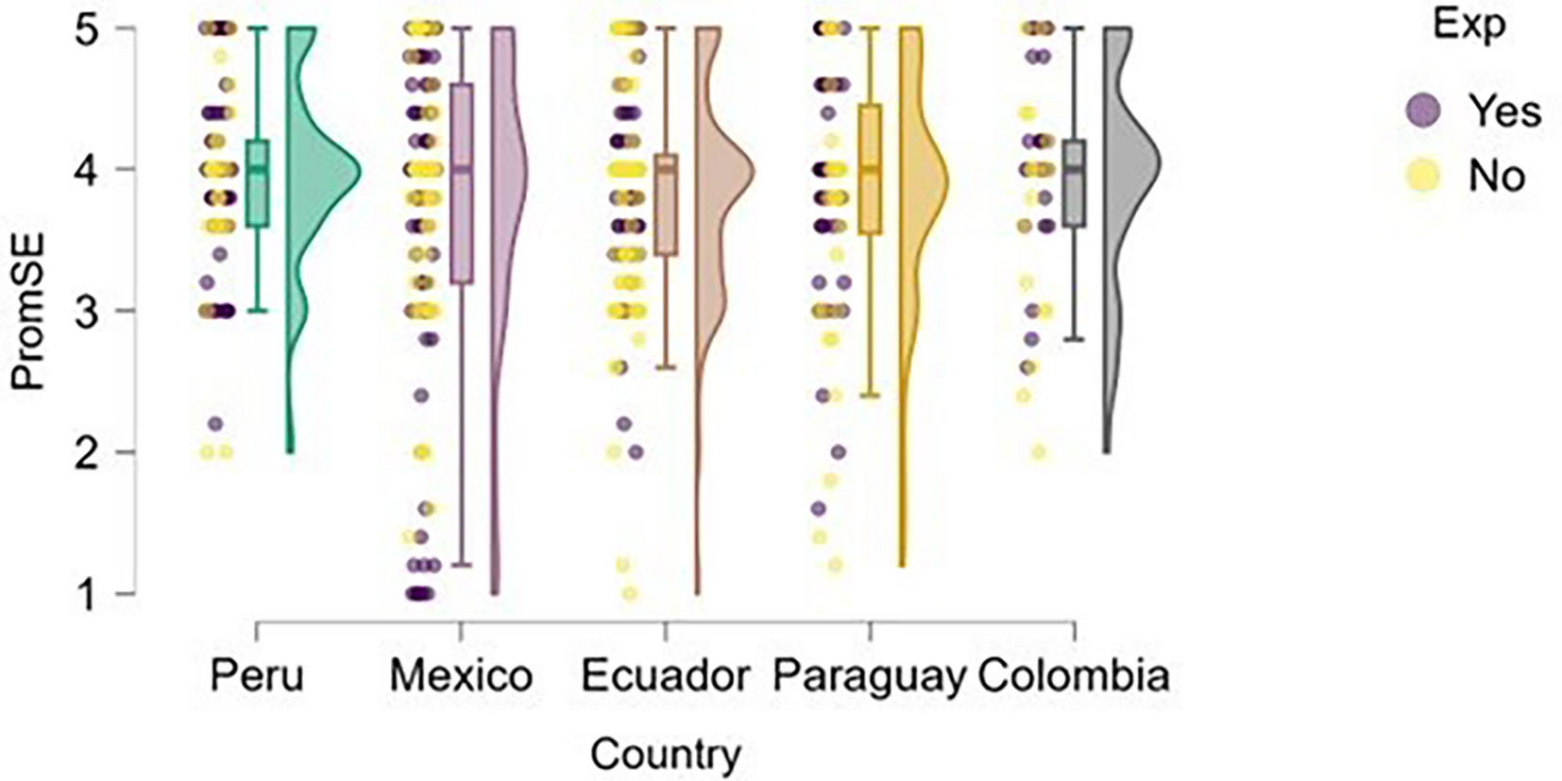
Entrepreneurial self-efficacy by entrepreneurial experience. Raincloud plots comparing self-efficacy between students with and without prior entrepreneurial experience, grouped by country. Scores on a 5-point Likert scale.

On the other hand, we examined whether actual entrepreneurial experience reinforced perceived self-efficacy within the study sample ( Figure 5). The figure suggests that entrepreneurial expertise is associated with higher levels of perceived self-efficacy across all countries represented. This finding is consistent with Bandura’s (1991) Social Cognitive Theory, which posits that personal experiences and performance in specific tasks strengthen individuals’ beliefs in their efficacy.

Peru and Mexico show a more apparent distinction between students with and without entrepreneurial experience: those with experience exhibit higher levels of self-efficacy. Ecuador, Paraguay, and Colombia also reflect this trend, although with a smaller gap between groups, suggesting that expertise still contributes, but may be interacting with other contextual factors.

### Cognitive load

**Figure 6.**
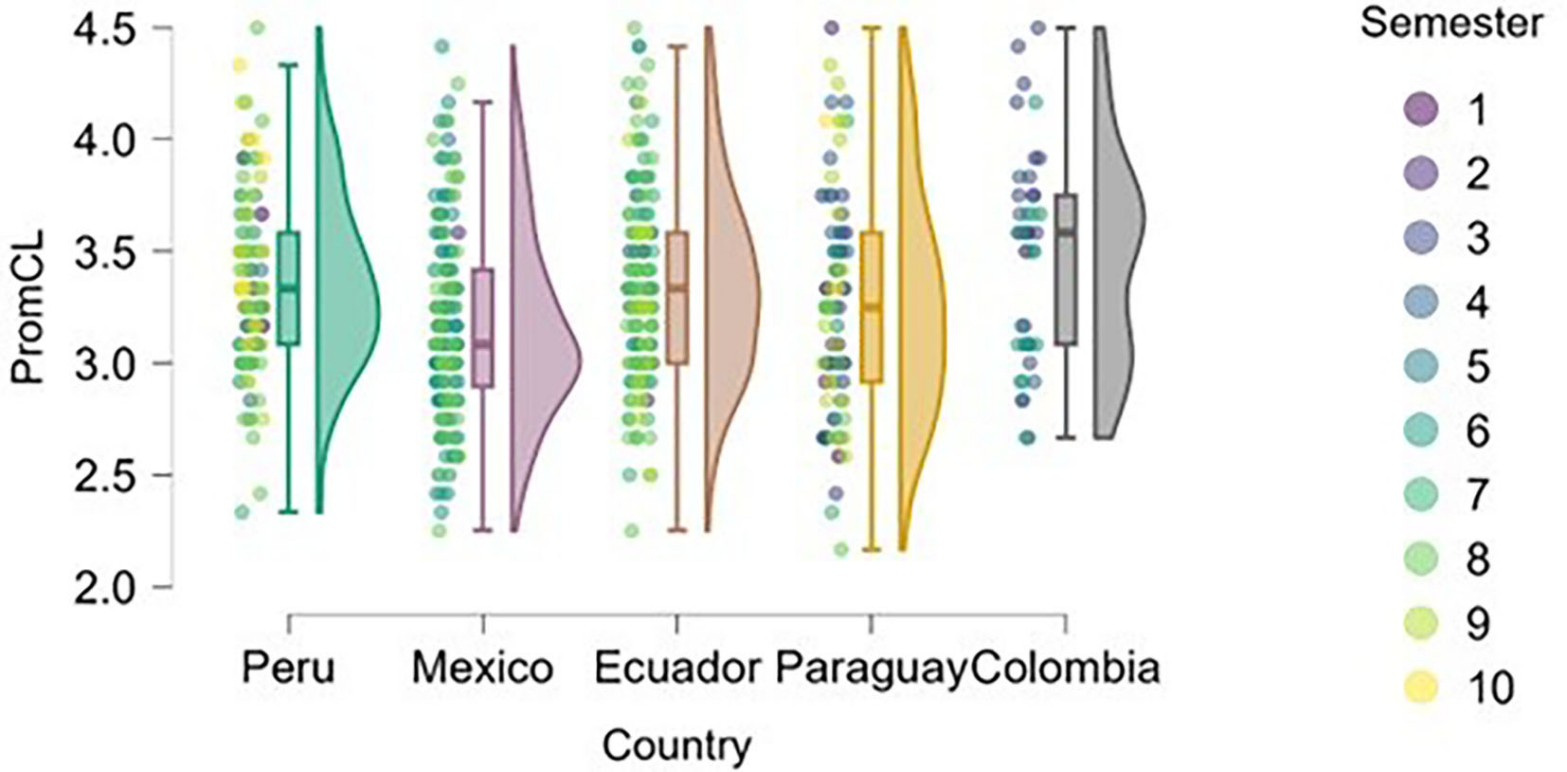
Cognitive load by academic semester and country. Raincloud plots showing cognitive load scores by semester for each country. Higher scores indicate greater perceived load. 5-point Likert scale.

On the other hand, we found that students in advanced semesters tend to experience higher cognitive load ( Figure 6), particularly in Peru, Mexico, and Paraguay. This is to be expected, as academic demands increase, extracurricular commitments and vocational decisions become more frequent, and personal and societal expectations regarding professional futures intensify.

Peru and Paraguay show greater dispersion across semesters, allowing for a more precise visualization of the progression of cognitive stress. In Ecuador and Colombia, the trend is less pronounced, possibly due to differences in curricular load or pedagogical approach.

### Emotional well-being

**Figure 7.**
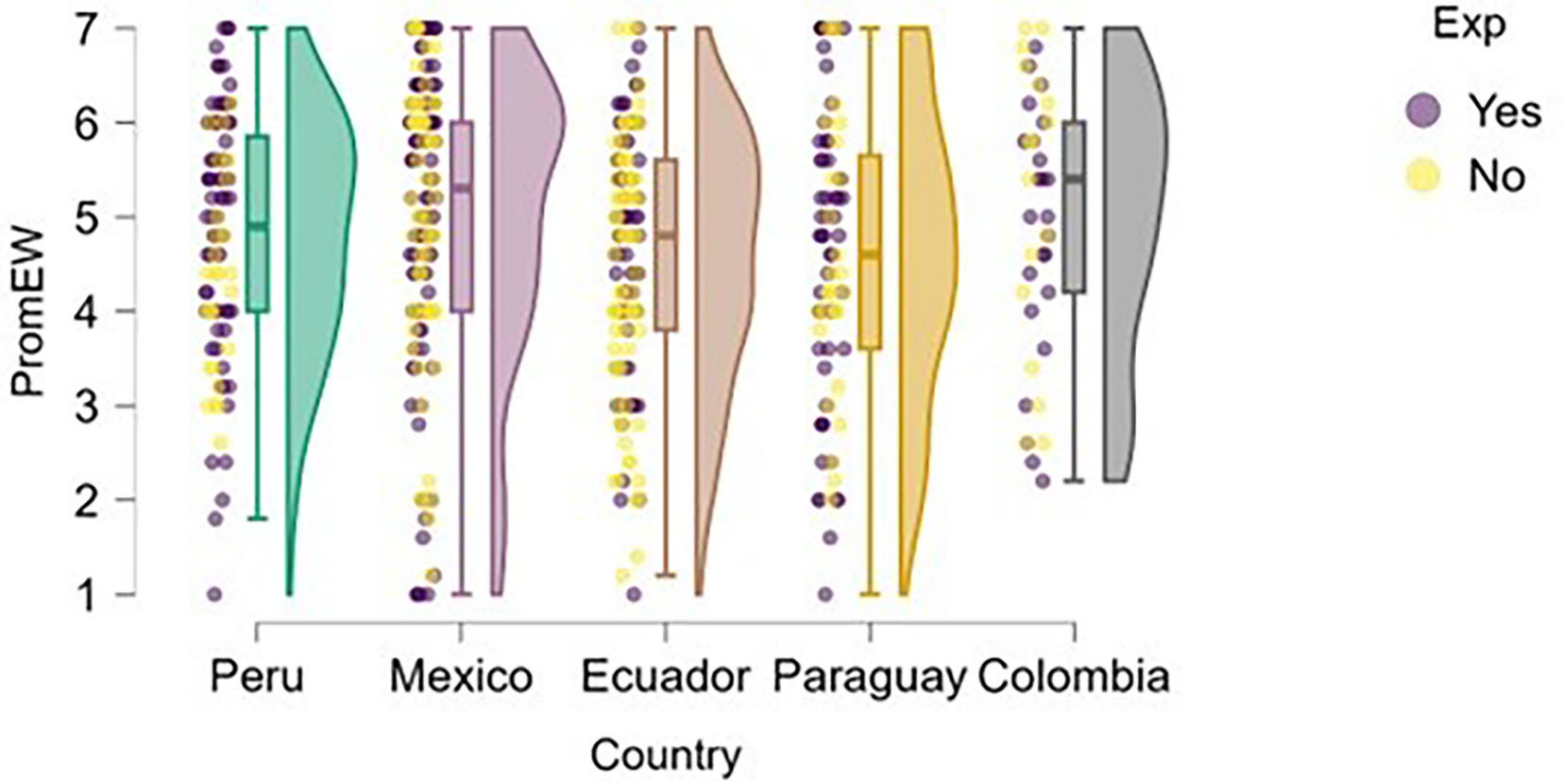
Emotional well-being by entrepreneurial experience. Raincloud plots comparing well-being between students with and without prior entrepreneurial experience. Scores on a 5-point Likert scale; higher scores indicate higher well-being.

Regarding students’ perceived emotional well-being, Figure 7 shows that, in most countries, students with prior entrepreneurial experience tend to report slightly higher or more consistent levels of well-being compared to those without experience. In Peru, students with entrepreneurial experience are more concentrated at higher levels of well-being, with lower dispersion than their inexperienced peers, which may be associated with greater self-fulfillment or a stronger sense of entrepreneurial purpose.

In Mexico and Ecuador, although there is some overlap, a slight advantage in well-being can also be observed among experienced students, although the differences are not pronounced. In Paraguay, the high dispersion in both groups is notable, especially among those without experience, which may reflect greater uncertainty or lack of direction. Finally, in Colombia, both groups show similar levels, although students with experience appear less likely to report very low levels of emotional well-being.

### Flow

**Figure 8.**
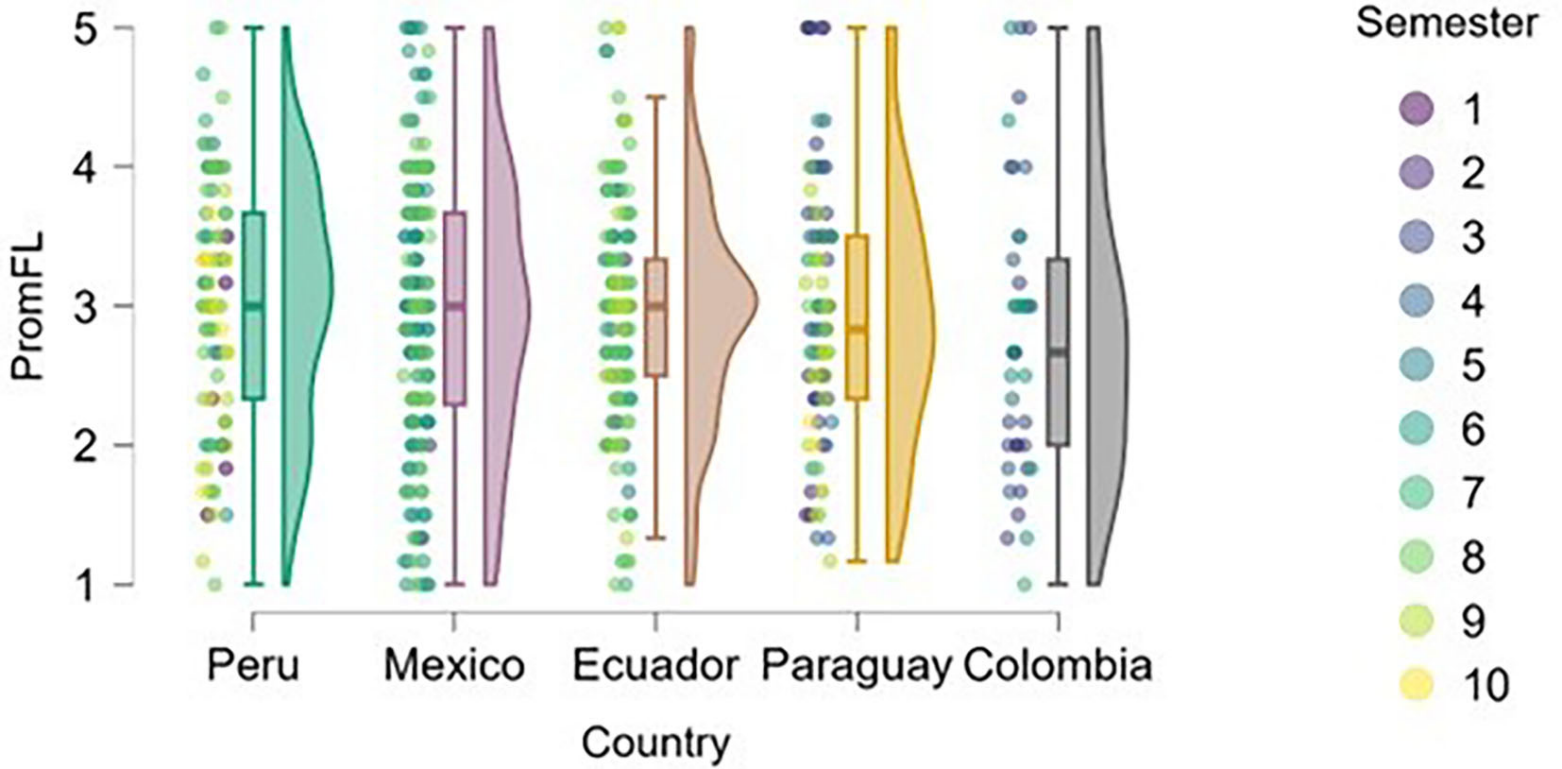
Flow levels by academic semester and country. Raincloud plots showing flow scores across semesters in each country. Scores on a 5-point Likert scale.

Figure 8 shows an upward trend in work-related flow across academic semesters, which can be interpreted as a progressive increase in students’ ability to organize tasks, maintain focus, and master content as they advance in their university education. This relationship appears to be more evident in Peru and Mexico, where students in higher semesters report higher levels of flow compared to those in earlier semesters.

In countries such as Ecuador, Paraguay, and Colombia, the relationship between semester and flow level appears less clear, with a wider dispersion of responses across different academic levels. This may be due to differences in pedagogical styles, institutional support, or curricular load, which warrants further examination.

### Emotional Competence

**Figure 9.**
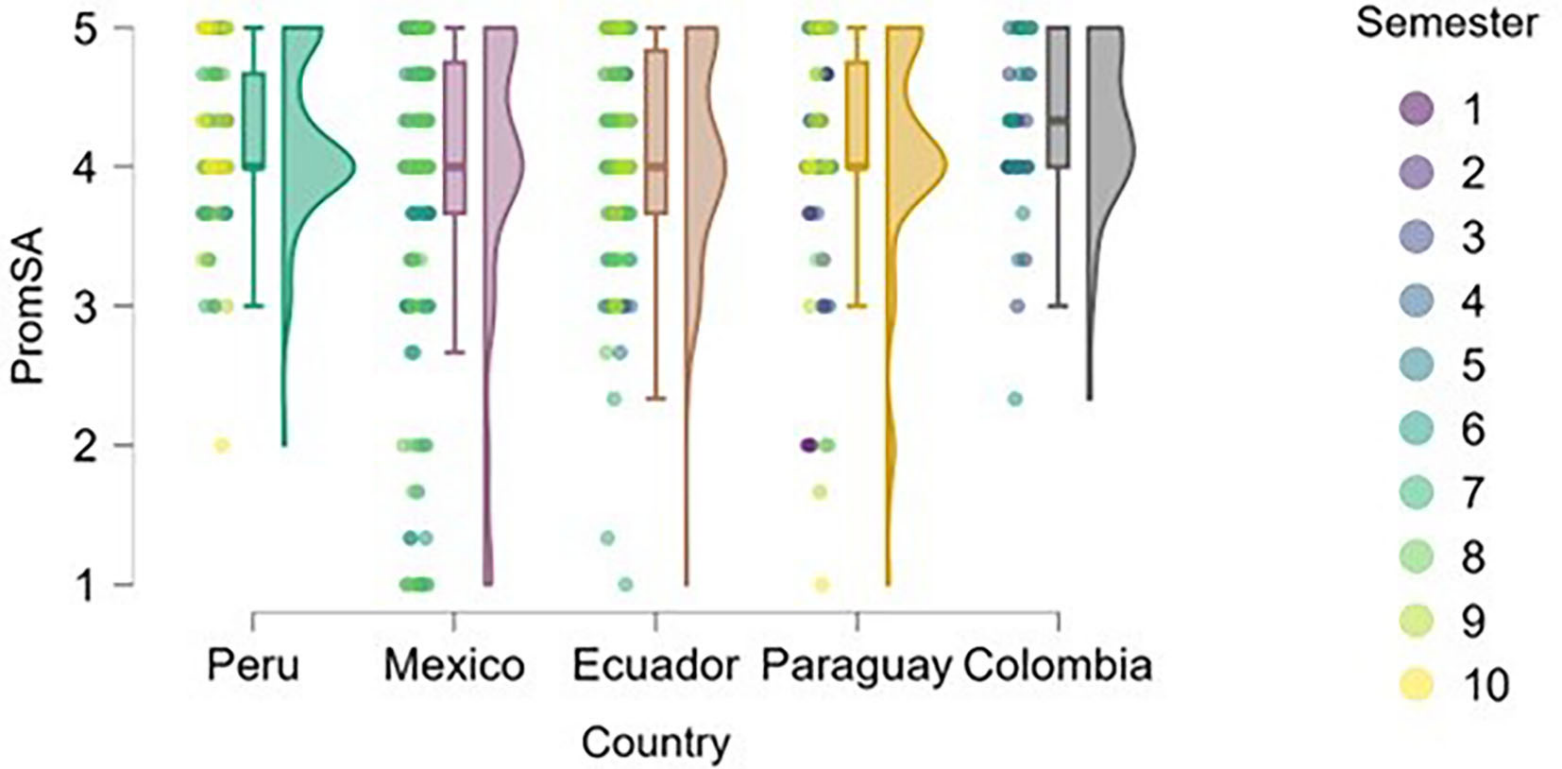
Self-awareness by academic semester and country. Raincloud plots showing self-awareness scores across semesters for each country. Scores on a 5-point Likert scale.

Emotional competence was examined across three dimensions: self-awareness, empathy, and social skills. In the case of self-awareness ( Figure 9), several countries—notably Mexico and Colombia—show a greater concentration of high scores in mid and advanced semesters, which may indicate that prolonged exposure to university experiences fosters the development of self-awareness.

In Peru and Ecuador, scores are more dispersed, but a slight upward trend is observed in advanced semesters. In Paraguay, while there is greater overall dispersion, self-awareness levels appear to remain relatively high even in the early semesters, which may be related to specific characteristics of the academic program or type of training provided.

**Figure 10.**
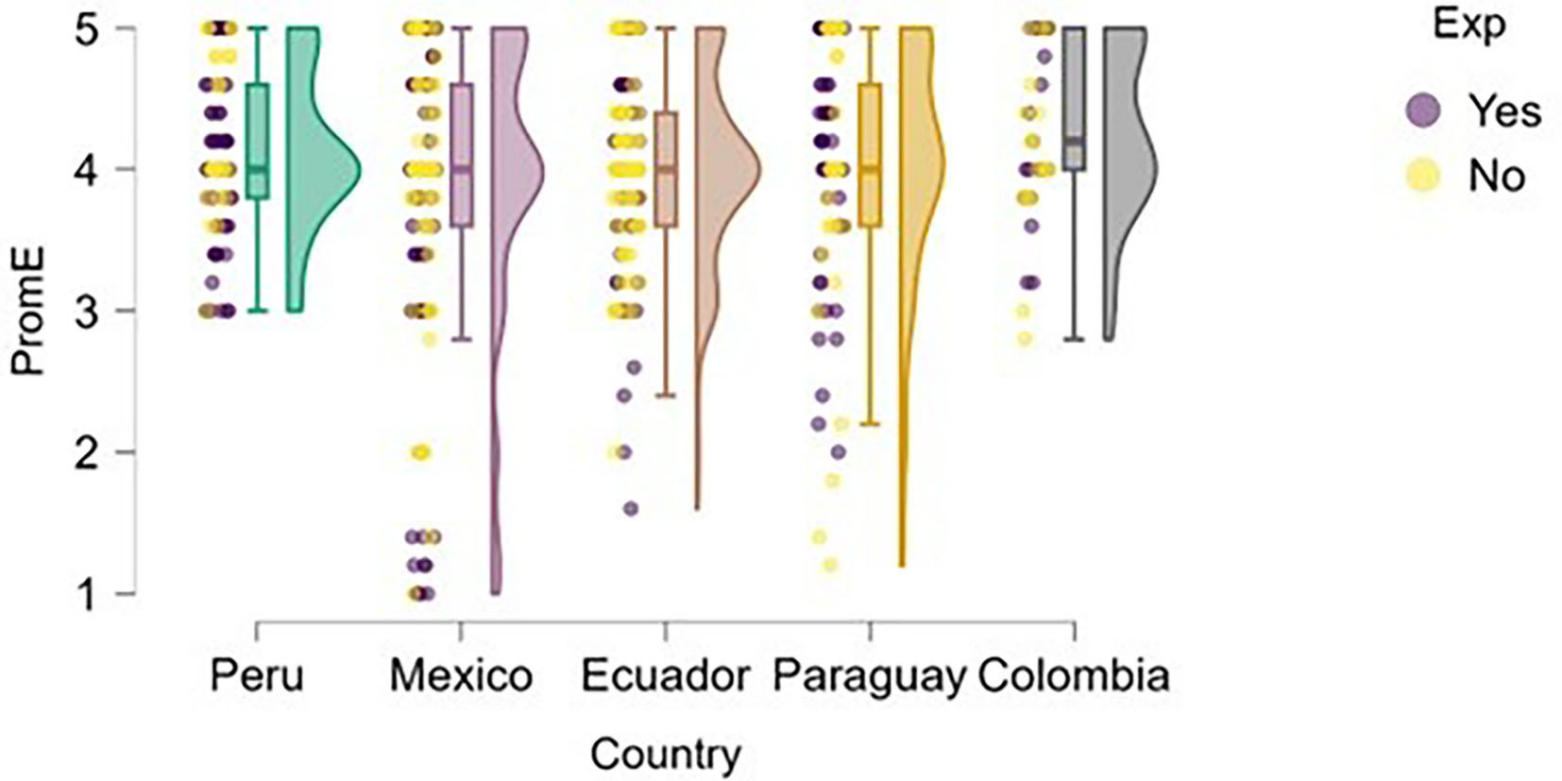
Empathy by entrepreneurial experience and country. Raincloud plots comparing empathy levels between students with and without entrepreneurial experience. Scores on a 5-point Likert scale.

Regarding empathy ( Figure 10), the data suggest that entrepreneurial experience is associated with slightly higher levels of empathy in several national contexts. This is particularly evident in Mexico, Paraguay, and Colombia, where participants with experience show score concentrations closer to the maximum. In Peru, empathy levels are generally high across all groups, with no notable difference between those with or without experience. In Ecuador, both groups show greater dispersion, although a slight advantage is observed among students with entrepreneurial experience.

This may be explained by the fact that entrepreneurship exposes students to situations where they must understand and anticipate the needs of others, develop effective communication, and collaborate—skills closely related to empathy.

**Figure 11.**
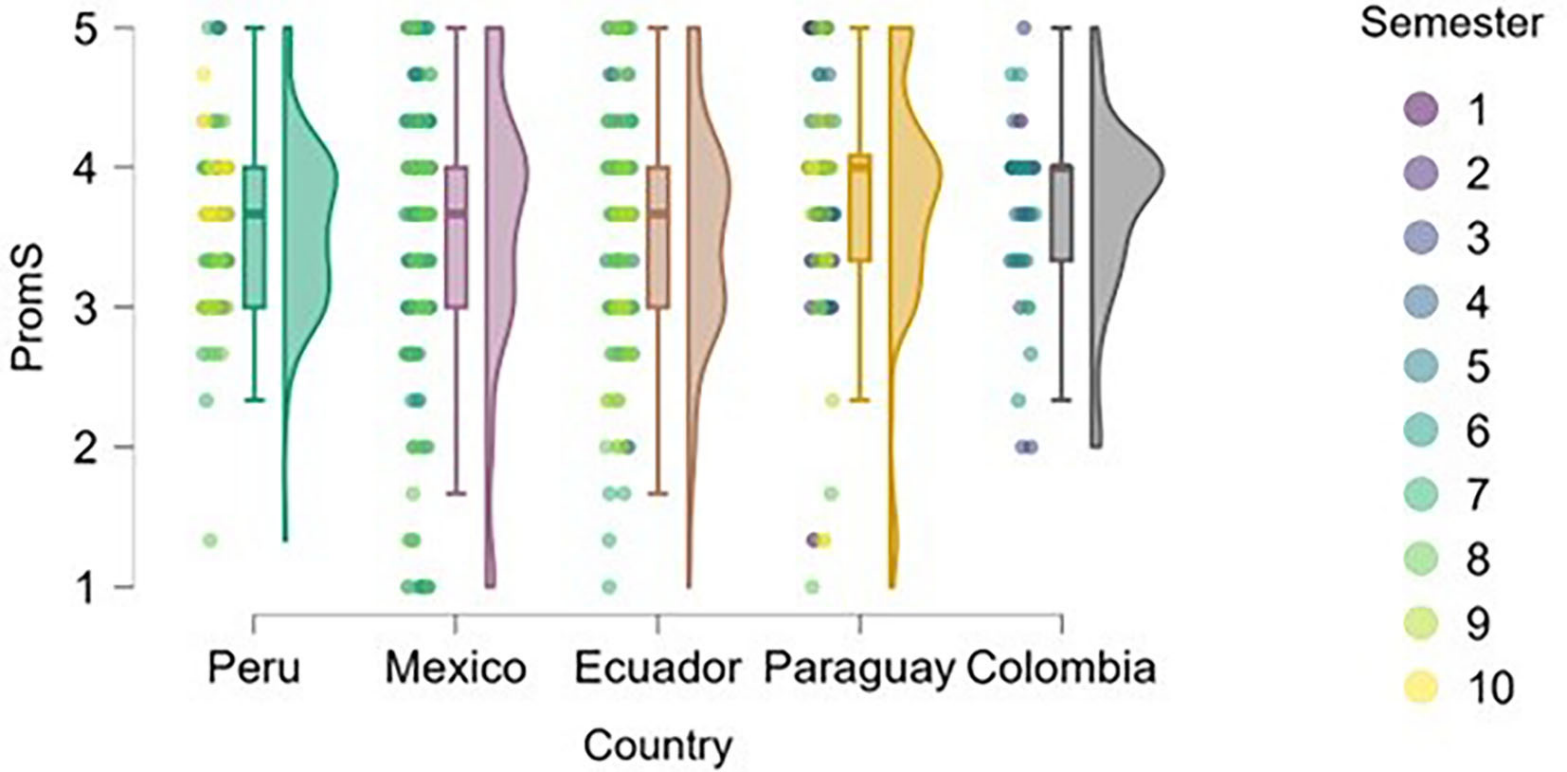
Social skills by academic semester and country. Raincloud plots showing social skills scores across semesters in each country. Scores on a 5-point Likert scale.

Regarding social skills ( Figure 11), although no drastic trend is observed, in some countries such as Peru and Colombia, there appears to be a slight positive association between academic semester and social skills. In other words, as students progress through their university education, they may be developing social skills more frequently—such as teamwork, leadership, and interpersonal relationship management—likely due to increased exposure to group experiences, presentations, internships, or collaborative projects.

However, this trend is not consistent across all countries, which may be due to curricular or institutional differences in the type of training and activities that foster these skills.

### Entrepreneurial action

**Figure 12.**
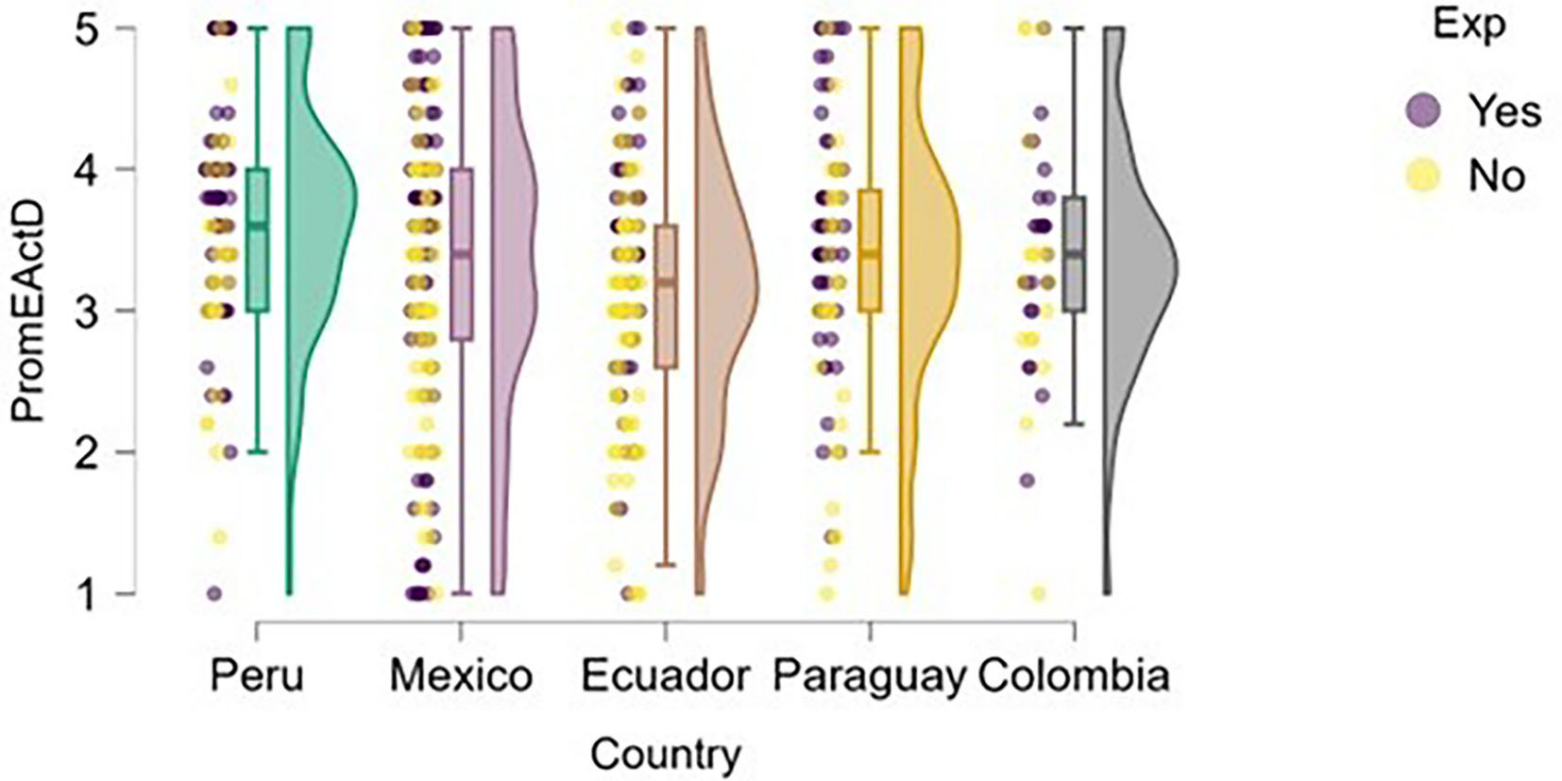
Entrepreneurial action – discovery phase by entrepreneurial experience. Raincloud plots showing discovery-phase entrepreneurial action scores for students with and without prior entrepreneurial experience, by country. Scores on a 5-point Likert scale.

Entrepreneurial action was assessed through two dimensions: discovery and exploitation. Regarding the discovery phase, Figure 12 suggests that students with prior entrepreneurial experience tend to exhibit higher levels of entrepreneurial action compared to those who have not previously engaged in entrepreneurship.

In countries such as Mexico, Ecuador, and Colombia, a greater concentration of high scores is observed among students with entrepreneurial experience. In Peru, although overall entrepreneurial action is high, the distribution is also elevated for the experienced group. In Paraguay, the difference is less pronounced, but the general trend remains consistent.

This pattern aligns with the work of authors such as McMullen & Shepherd (2006), who argue that prior experience facilitates the identification of new opportunities, as it helps develop entrepreneurial alertness and provides familiarity with environmental challenges.

**Figure 13.**
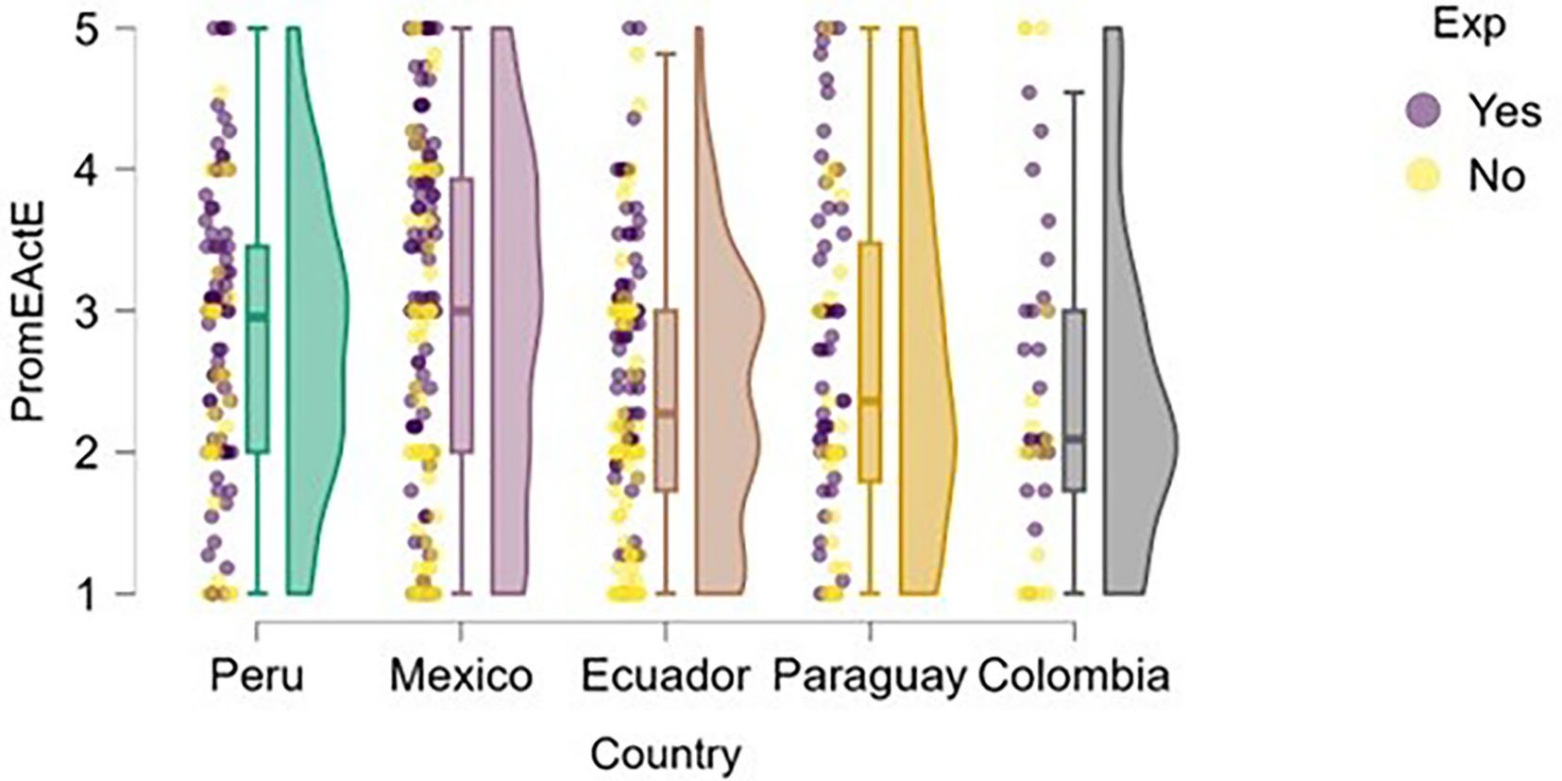
Entrepreneurial action – exploitation phase by entrepreneurial experience. Raincloud plots showing exploitation-phase entrepreneurial action scores for students with and without prior entrepreneurial experience, by country. Scores on a 5-point Likert scale.

A similar finding emerges in the exploitation dimension ( Figure 13), where students with entrepreneurial experience tend to report slightly higher levels of entrepreneurial action—particularly in Mexico and Ecuador. However, in countries like Paraguay and Colombia, the differences are much less pronounced, and both distributions (with and without experience) show greater dispersion and overlap.

In the case of Peru, although students with entrepreneurial experience show higher scores, there is also a group without experience that exhibits similarly high levels. This may indicate that the educational environment or local entrepreneurial ecosystem supports the transition to the exploitation phase even without prior experience.

This finding is consistent with the propositions of Shane and Venkataraman (2000), who suggest that entrepreneurial action in the exploitation phase depends not only on opportunity recognition but also on access to resources, prior knowledge, and institutional support. Likewise, previous experience may serve as a facilitator by enhancing confidence and enabling more effective use of resources in the transition from discovery to exploitation ( McMullen & Shepherd, 2006).

### Financial knowledge

**Figure 14.**
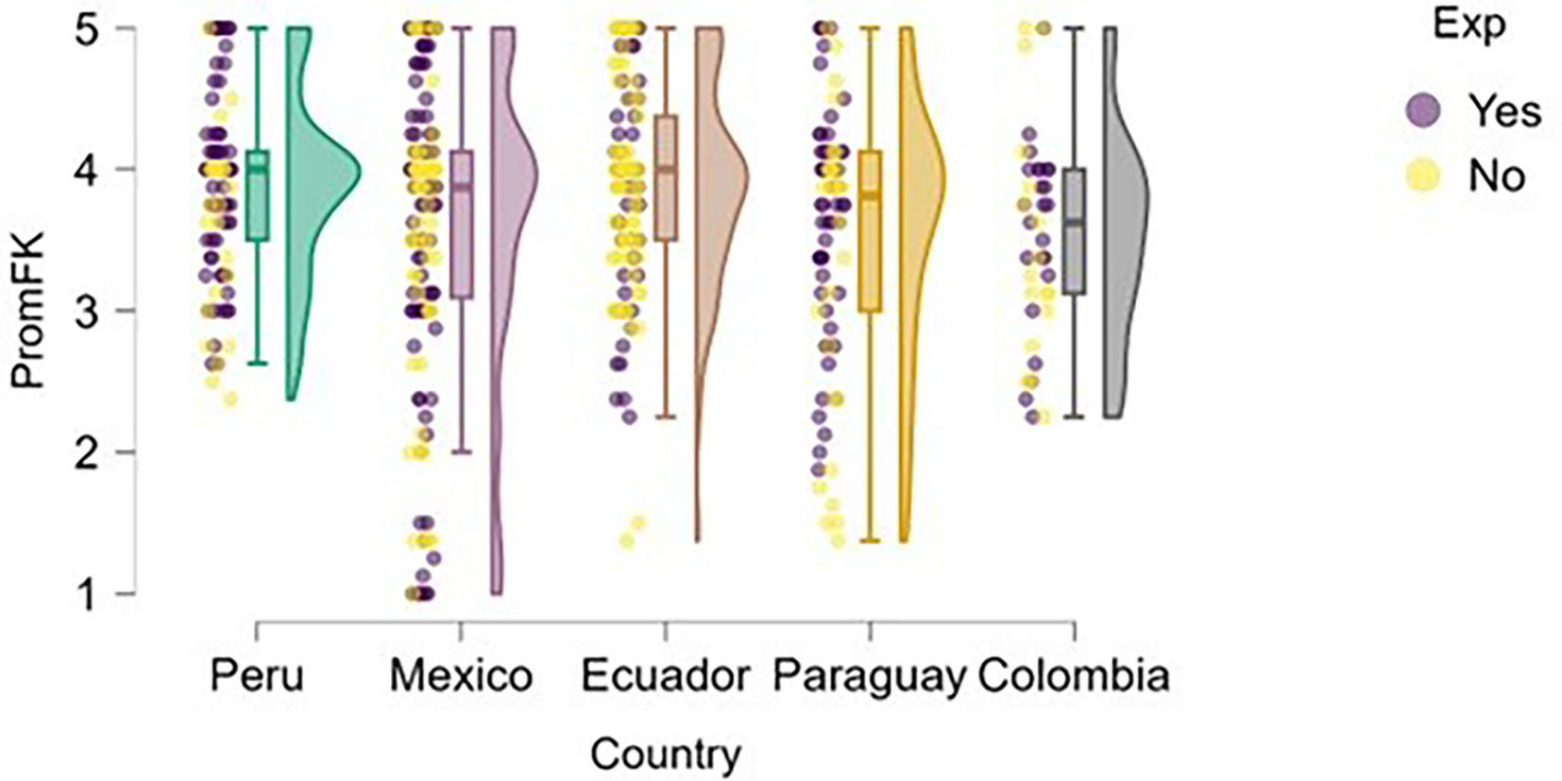
Financial knowledge by entrepreneurial experience and country. Raincloud plots comparing financial knowledge scores between students with and without entrepreneurial experience. Higher scores indicate better financial knowledge.

Finally, regarding financial knowledge ( Figure 14), Peru presents the highest and most consistent levels among the countries analyzed, with a median close to 4.5 and a distribution concentrated in the upper range of the scale. Both students with entrepreneurial experience and those without it tend to perform well on this variable, suggesting a potentially more effective integration of financial content into the Peruvian university curriculum.

In contrast, Mexico shows greater dispersion in responses, including cases with very low levels of financial knowledge, which may reflect disparities in training or access to basic economic content. Ecuador, Paraguay, and Colombia show moderate medians (between 3.5 and 4), but also higher variability—especially in Paraguay—possibly indicating gaps in formal financial education or limited practical exposure to fundamental economic concepts.

***Results of differences between countries***

Since the variables did not meet the assumptions of normality or homogeneity of variances, the non-parametric Kruskal-Wallis test was used to determine whether significant differences existed among the five participating countries (Peru, Mexico, Ecuador, Paraguay, and Colombia) in key variables related to university entrepreneurship. Of the 18 variables analyzed, nine showed statistically significant differences (p < 0.05), as summarized in Table 1.

**Table 1.** Variables with statistically significant differences between countries.

Variable	χ ^2^	p-value	Cross-country significant differences
PromU	33.82	0.001	Mexico vs. Peru, Ecuador, Colombia, Paraguay
PromEActD	19.07	0.001	Mexico vs. Ecuador, Paraguay
PromEActE	18.35	0.001	Mexico vs. Ecuador, Paraguay
PromEI	16.08	0.003	Peru > Mexico, Peru > Ecuador
PromCL	29.13	0.001	Mexico > Ecuador, Colombia
PromEW	13.83	0.008	Mexico > Peru, Mexico > Paraguay
PromFK	11.17	0.025	Mexico > Paraguay
PromR	10.99	0.027	Mexico > Ecuador, Ecuador < Paraguay
PromSS	9.75	0.045	Diferencia global

Results of the Kruskal–Wallis test for the five participating countries (Mexico, Peru, Ecuador, Paraguay, and Colombia). Variables with p < 0.05 are shown with χ ^2^ statistic, p-value, and significant pairwise differences based on Dunn’s post-hoc test with corrections.

The results clearly show that Mexico ranks highest in several key dimensions, particularly in university training, financial knowledge, entrepreneurial action, and emotional well-being. Peru also stands out for exhibiting the highest levels of entrepreneurial intention compared to the other countries. In contrast, Ecuador and Paraguay tend to display lower scores across multiple dimensions, suggesting potential gaps in the development of the university entrepreneurial ecosystem.

***Correlations with entrepreneurial intention and action***

To assess the factors associated with entrepreneurial behavior, Spearman’s rank correlation (ρ) was applied, allowing for the identification of the strength and direction of associations between ordinal variables without assuming normality. The results reveal consistent associations between entrepreneurial intention (PromEI) and key variables of the entrepreneurial profile. Entrepreneurial attitude (PromEA) shows the strongest correlation with PromEI (ρ = 0.664, p < .001), followed by self-efficacy (PromSE, ρ = 0.370), entrepreneurial action in the discovery phase (PromEActD, ρ = 0.371), and perceived university support (PromU, ρ = 0.288). These findings suggest that strengthening personal beliefs, attitudes, and institutional support is linked to higher levels of entrepreneurial intention.

Regarding entrepreneurial action in the discovery phase (PromEActD), it is strongly associated with the exploitation phase (PromEActE, ρ = 0.642), self-efficacy (PromSE, ρ = 0.507), financial knowledge (PromFK, ρ = 0.397), and self-awareness (PromSA, ρ = 0.353). These relationships suggest that students who are already acting on opportunities also tend to discover new ones, particularly when they feel capable and possess the cognitive and emotional tools to evaluate their environment.

Finally, in the exploitation phase (PromEActE), in addition to its strong association with the discovery phase (ρ = 0.642), significant correlations were observed with flow (PromFL, ρ = 0.379), self-efficacy (PromSE, ρ = 0.283), emotional well-being (PromEW, ρ = 0.275), and financial knowledge (PromFK, ρ = 0.252). These results reinforce the idea that successfully executing entrepreneurial projects requires not only technical preparation but also emotional stability and personal confidence.

***Regression models for entrepreneurial intention and action***

The multiple linear regression models were statistically significant and explained a substantial proportion of variance in their respective dependent variables. The model with PromEI (entrepreneurial intention) as the dependent variable showed a good fit (F(5, 534) = 133.72, p < .001), explaining 55.2% of the variance (adjusted R ^2^ = 0.552). The model for PromEActD (entrepreneurial action – discovery) showed the highest fit (F(3, 536) = 269.22, p < .001), accounting for 59.9% of the variance (adjusted R ^2^ = 0.599). Meanwhile, the model for PromEActE (entrepreneurial action – exploitation) was also significant (F(5, 534) = 26.14, p < .001), explaining 27.3% of the variance (adjusted R ^2^ = 0.273). In all cases, the classical assumptions of linear regression were verified, and no multicollinearity issues were identified.

**Figure 15.**
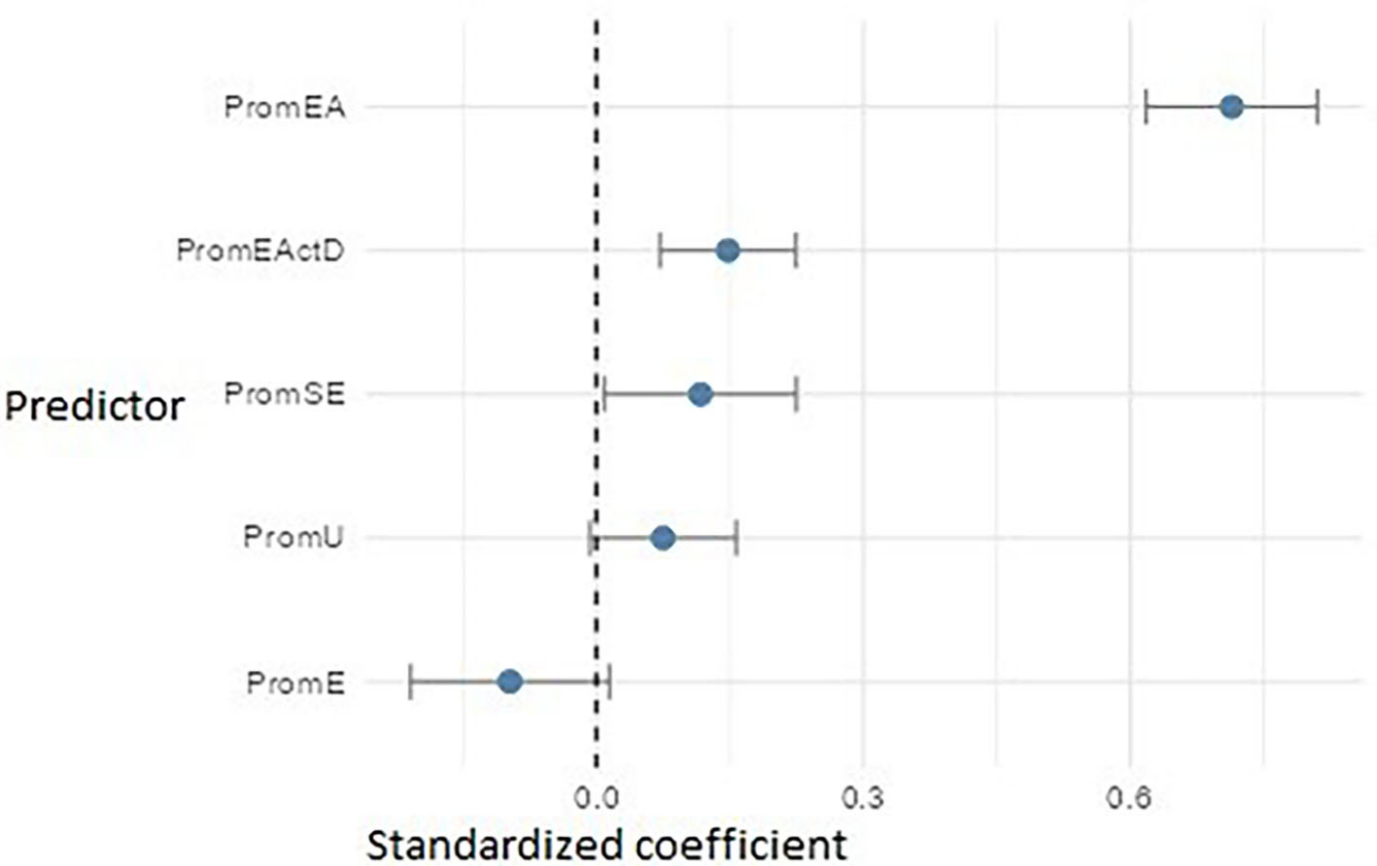
Multiple linear regression model predicting entrepreneurial intention. Path diagram showing standardized beta coefficients for predictors of entrepreneurial intention (PromEI).

In the PromEI model ( Figure 15), entrepreneurial attitude (PromEA) emerged as the strongest predictor (β ≈ 0.71), highlighting its key role as a transversal formative axis. It was followed by entrepreneurial action – discovery (PromEActD) (β ≈ 0.14) and self-efficacy (PromSE) (β ≈ 0.12), underscoring the importance of teaching students how to identify real opportunities and build entrepreneurial confidence. University support (PromU) also had a small but significant effect (β ≈ 0.08), indicating that the institutional environment should be reinforced. Finally, empathy (PromE) showed an adverse impact (β ≈ –0.10), possibly suggesting that in specific contexts, high levels of empathy may be associated with lower entrepreneurial intention—perhaps due to risk aversion or a less autonomous orientation.

**Figure 16.**
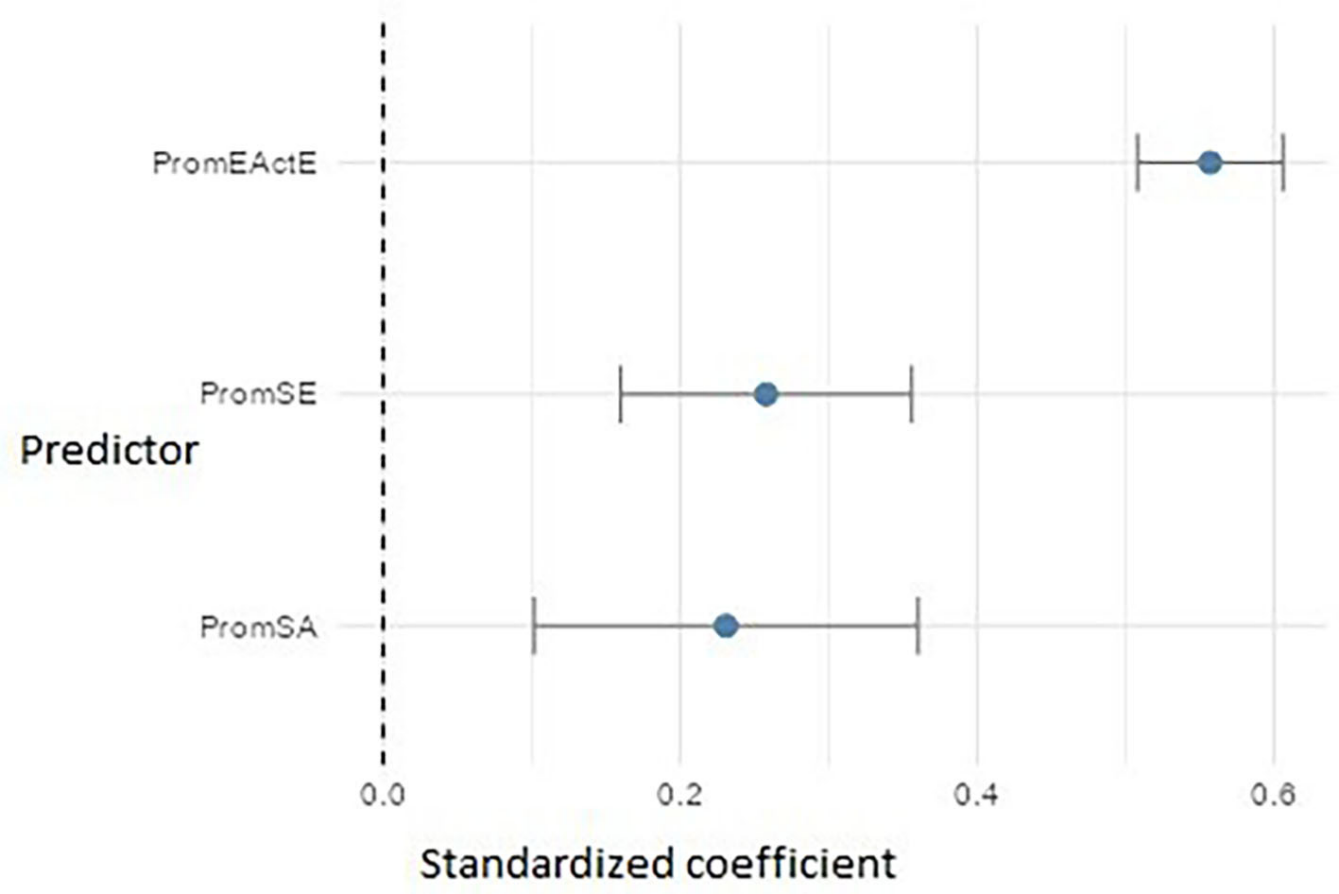
Regression model for entrepreneurial action – discovery phase. Path diagram showing significant predictors of discovery-phase entrepreneurial action (PromEActD).

For PromEActD ( Figure 16), the three significant predictors were: entrepreneurial action – exploitation (PromEActE) (β ≈ 0.56), which emerged as the strongest factor, suggesting that teaching students how to execute projects enhances their ability to discover opportunities. Self-efficacy (PromSE) (β ≈ 0.26) and self-awareness (PromSA) (β ≈ 0.23) also showed significant effects, highlighting the importance of cultivating both personal confidence and the ability to recognize and regulate one’s own emotions and strengths during the early stages of the entrepreneurial process.

**Figure 17.**
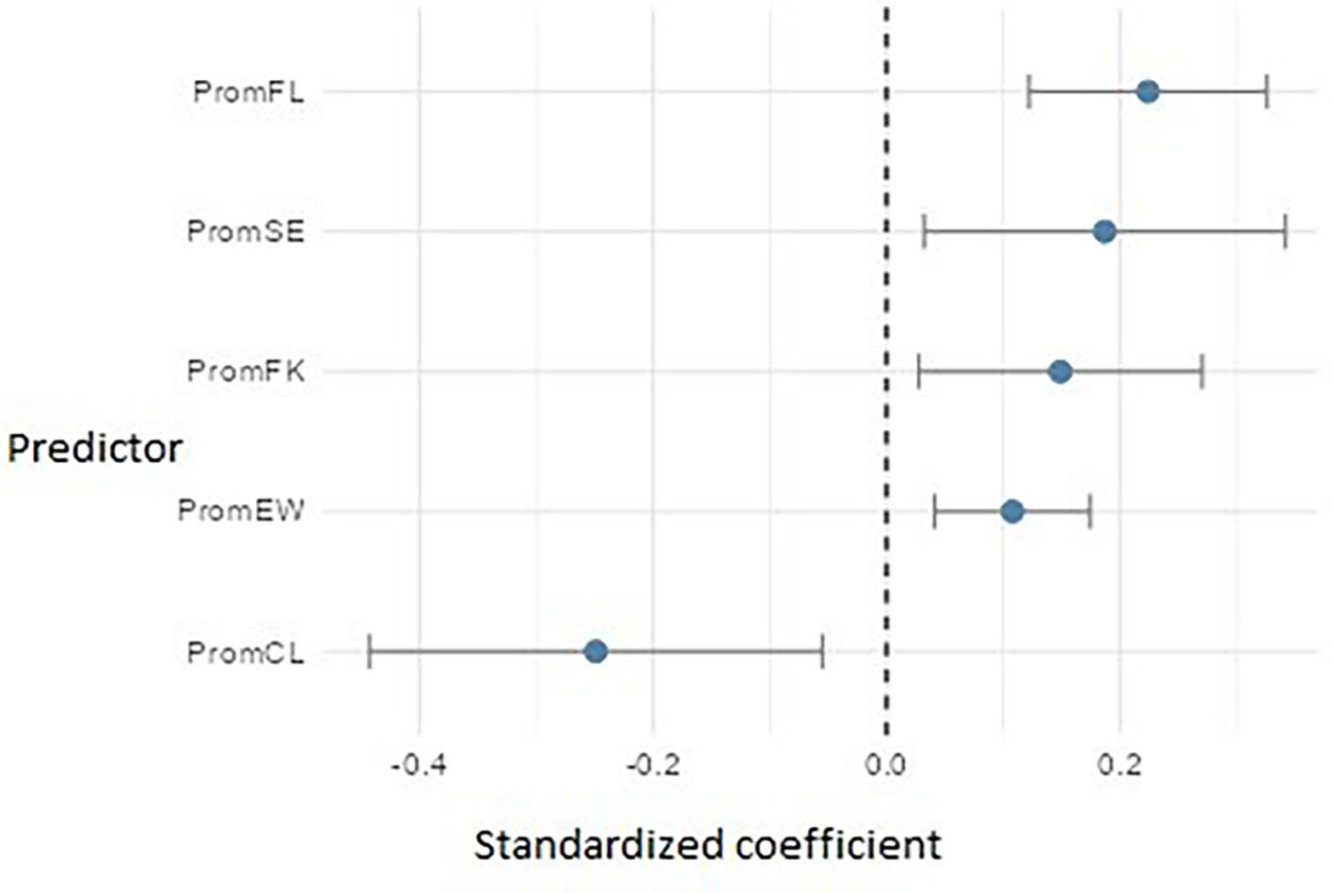
Regression model for entrepreneurial action – exploitation phase. Path diagram showing significant predictors of exploitation-phase entrepreneurial action (PromEActE).

Regarding PromEActE ( Figure 17), the model revealed five significant predictors. Flow (PromFL) stood out with a relevant positive effect (β ≈ 0.22), followed by self-efficacy (PromSE) (β ≈ 0.19), practical financial knowledge (PromFK) (β ≈ 0.15), and emotional well-being (PromEW) (β ≈ 0.11), pointing to the importance of preparing students who are not only technically skilled but also emotionally balanced. However, cognitive load (PromCL) showed an adverse effect (β ≈ = -0.25), which may indicate that excessive mental demands or academic overload interfere with students’ ability to carry out entrepreneurial actions.

### Recommendations

Based on the study’s findings, the following recommendations are proposed to strengthen the university’s entrepreneurial support ecosystem. These recommendations integrate strategic institutional aspects as well as curricular and operational components of academic programs, acknowledging the influence of contextual, emotional, and cognitive variables that vary across countries.

*Strategic Recommendations (Institutional and Curricular Level)*

The cross-country differences in entrepreneurial intention, attitude, and motivation highlight the need for adaptive curricula that are responsive to students’ cultural and emotional profiles. In Ecuador, it is recommended to include activities that promote proactivity and entrepreneurial vision, such as social innovation projects or entrepreneurship challenges. In Mexico, emotional well-being and self-regulation could be incorporated transversally into the entrepreneurship curriculum through reflective practices or stress management workshops.

Given that empathy, self-efficacy, and motivation were highly correlated with entrepreneurial intention, it is advisable to integrate these competencies as explicit learning outcomes in key courses. This can be achieved through active methodologies such as project-based learning, role-plays, ethical decision-making simulations, cooperative activities, and peer feedback and self-assessment sessions.

The impact of social support on both entrepreneurial intention and action justifies the design of collaborative training spaces such as entrepreneurship labs or academic incubators, where interaction, teamwork, tolerance for failure, and communities of practice are fostered. These spaces could be aligned with academic credit or incorporated into practical courses.

The finding that cognitive load negatively affects the entrepreneurial exploitation phase (β = –0.25) suggests the need to revise the sequencing and density of entrepreneurship courses, avoiding their concentration during high-demand semesters. It is recommended to incorporate into the curriculum short sessions on mindfulness, study techniques, and emotional regulation—or even active break weeks—to help prevent cognitive and emotional exhaustion.

Moreover, regression results showed that entrepreneurial attitude (β = 0.71) and self-efficacy (β ≈ 0.12–0.26) are the most relevant predictors of entrepreneurial intention and action. Therefore, it is essential to reinforce these variables across the curriculum through leadership workshops, entrepreneurial storytelling, practical challenges, and spaces for individualized feedback. In addition, self-awareness—which showed a positive effect in early stages—should be promoted through reflective portfolios and metacognitive exercises.

*Operational Recommendations (Entrepreneurship Programs and Student Support)*

It is recommended to offer personalized training pathways according to the student’s stage in the entrepreneurial process:

• For those in the discovery phase, include modules on locus of control, creative thinking, design thinking, and environmental analysis.
• For those in the exploitation phase, offer courses or workshops on personal finance, resource management, adaptive leadership, and uncertainty management. This is consistent with the findings that identified financial knowledge (β ≈ 0.22) and its practical application (β ≈ 0.15) as significant predictors of the exploitation phase.

The correlations found between self-regulation, motivation, and self-awareness support the development of diagnostic tools to be applied at the beginning of the academic cycle (e.g., surveys on emotional competencies and entrepreneurial profiles). These results can be used to assign tailored mentoring, suggest electives, and personalize students’ learning pathways. Additionally, it is recommended to monitor indicators of cognitive load and emotional well-being during the later stages of academic training.

It is also advisable to include mentorship spaces with entrepreneurial alums or emotional coaching sessions led by professionals, focused on strengthening self-efficacy, resilience, and sense of purpose. These activities can be formally integrated into capstone projects or supervised internships, with the possibility of earning academic credit.

Finally, implement continuous evaluation systems that assess not only academic outcomes but also the evolution of emotional well-being, perceived institutional support, and the progression of the entrepreneurial profile. This information will support curriculum refinement, adjustment of academic workload, and better targeting of institutional resources.

## Ethical approval

This study was reviewed and approved by the Comité Institucional de Bioética of the Fundación Universitaria Konrad Lorenz (Bogotá, Colombia) during Session No. 34 held on August 15, 2024, under the project title “Impact of Cognitive Load and Emotional Well-being on the Entrepreneurial Intention of Business School Students Belonging to the Accreditation Council for Business Schools and Programs”. Approval status: Approved. All participants gave written informed consent before participation. Institutional protocols and the principles of the Declaration of Helsinki conducted the study.

## AI use disclosure

I have read and agree to comply with the F1000 AI Policy. I confirm that during the preparation of this manuscript, I used ChatGPT (OpenAI, GPT-4, version August 2025) exclusively to assist with the translation of the original Spanish text into English. The content was subsequently reviewed and edited by the authors to ensure accuracy and clarity.

## Data Availability

Zenodo: Entrepreneurial Intention and Action in ACBSP-accredited Latin American Universities (2024–2025). https://doi.org/10.5281/zenodo.16945449 ( Alba et al., 2025). This project contains the following underlying data:
• Data.xlsx (anonymized Excel dataset containing responses from undergraduate students in Mexico, Peru, Ecuador, Paraguay, and Colombia on psychological, cognitive, and contextual variables related to entrepreneurial intention and action) Zenodo: Entrepreneurial Intention and Action in ACBSP-accredited Latin American Universities (2024–2025). https://doi.org/10.5281/zenodo.16945449 ( Alba et al., 2025). This project contains the following extended data:
• Spanish Questionnaire.pdf (final version of the survey instrument applied in the study) • English Questionnaire.pdf (final version of the survey instrument applied in the study) No reporting guidelines are associated with this article. Data is available under the terms of the Creative Commons Attribution 4.0 International license (CC-BY 4.0).
