# Supplementary material for: Cross-national Dataset on Psychological, Cognitive, and Contextual Factors of Entrepreneurial Behavior in Business Students
Source: F1000Res. 2026 Feb 14;14:935. Originally published 2025 Sep 18. [Version 3] doi: 10.12688/f1000research.168958.3 (PMC12982988; doi:10.12688/f1000research.168958.3)
Supplement: Peer review history

# Reviewer report: Reviewer response for version 3

**DOI:** 10.5256/f1000research.196266.r458599

**Published:** 2026-03-12

**Authors:** Ahmet Maslakçı

**Affiliations:** 1 World Peace University, Mersin, Nicosia, Turkey

Thank you for the revisions to the study.The authors had implemented the corrections I had suggested for their work.

Is the work clearly and accurately presented and does it cite the current literature?

Partly

If applicable, is the statistical analysis and its interpretation appropriate?

Partly

Are all the source data underlying the results available to ensure full reproducibility?

Yes

Is the study design appropriate and is the work technically sound?

Partly

Are the conclusions drawn adequately supported by the results?

Partly

Are sufficient details of methods and analysis provided to allow replication by others?

Partly

Reviewer Expertise:

Entrepreneurship-Multiculturalism-Organisational Behaviour

I confirm that I have read this submission and believe that I have an appropriate level of expertise to confirm that it is of an acceptable scientific standard.

# Reviewer report: Reviewer response for version 3

**DOI:** 10.5256/f1000research.196266.r458600

**Published:** 2026-03-05

**Authors:** Alejandro Valencia-Arias

**Affiliations:** 1 Universidad Señor de Sipán, Chiclayo, Peru

I confirm that the authors have adequately addressed the comments and suggestions raised during the review process. The revisions have improved the clarity, methodological transparency, and overall coherence of the manuscript. Therefore, I consider that the authors have satisfactorily complied with the requested modifications and the manuscript can be indexed.

Is the work clearly and accurately presented and does it cite the current literature?

Partly

If applicable, is the statistical analysis and its interpretation appropriate?

Yes

Are all the source data underlying the results available to ensure full reproducibility?

Yes

Is the study design appropriate and is the work technically sound?

Partly

Are the conclusions drawn adequately supported by the results?

Yes

Are sufficient details of methods and analysis provided to allow replication by others?

Partly

Reviewer Expertise:

Entrepreneurship

I confirm that I have read this submission and believe that I have an appropriate level of expertise to confirm that it is of an acceptable scientific standard.

# Response

**Published:** 2026-03-05

**Authors:** Marisleidy Alba

Dear Reviewer,

 Thank you very much for the time and careful attention you devoted to our manuscript. We sincerely appreciate your constructive comments and suggestions throughout the review process, which helped us to strengthen the work substantially.

 We are grateful for your confirmation that the revisions have improved the manuscript’s clarity, methodological transparency, and overall coherence, and that we have satisfactorily addressed the requested modifications. We also appreciate your indication that the manuscript can proceed to indexing.

 Thank you again for your valuable contribution.

 Kind regards,

 Marisleidy Alba

 (on behalf of all co-authors)

# Reviewer report: Reviewer response for version 2

**DOI:** 10.5256/f1000research.191969.r445561

**Published:** 2026-01-15

**Authors:** Ahmet Maslakçı

**Affiliations:** 1 World Peace University, Mersin, Nicosia, Turkey

The theoretical part of the study needs further development. The authors need to address the study's importance, its purpose, and its contribution to the literature and to practical life in greater detail. Although the rain cloud graphics method used in the study appears visually appealing, I would personally find it more satisfying if the study had been conducted using SPSS and Amos. The study's sample also requires a more detailed examination of how and why this sample was selected.

  

1. **Is the study design appropriate and is the work technically sound?** *The abstract section of the study should provide information on the purpose, methods, findings, and significance of the study. The introduction section of the study should mention why the variables were selected, the importance of the research question and how it differs from other studies. It should also mention the contributions the study will make to the literature and practical life. Studies conducted over the last 15 years are mentioned, but no references are provided or discussed. The theoretical background of the study should include the elements that should be in the introduction. The theoretical background should also describe the relationship among the variables, with references to relevant literature.*
2. **Are sufficient details of methods and analysis provided to allow replication by others?** *Information must be provided about the universities in Mexico, Peru, Ecuador, Paraguay and Colombia included in the study and the universities from which participants were selected. It would also be useful to include the study's conceptual model.*
3. **If applicable, is the statistical analysis and its interpretation appropriate?** *Although the use of rain cloud graphics is visually successful and innovative, using SPSS or Amos to present the explanatory graphics of this study and examine the effects of mediation and moderation would have made the model more understandable, given the selected variables and sample.*
4. **Are the conclusions drawn adequately supported by the results?** *Separating the Results and Discussion sections will enhance the study. This will ensure that the findings, evaluations, and analyses in the Discussion section are clearer and easier to understand. Additionally, the findings should be considered in terms of their contributions to the literature and their practical contributions.* *This will enable recommendations to be made to policymakers and university management. Future recommendations can also include cultural differences in the study.*

Is the work clearly and accurately presented and does it cite the current literature?

Partly

If applicable, is the statistical analysis and its interpretation appropriate?

Partly

Are all the source data underlying the results available to ensure full reproducibility?

Yes

Is the study design appropriate and is the work technically sound?

Partly

Are the conclusions drawn adequately supported by the results?

Partly

Are sufficient details of methods and analysis provided to allow replication by others?

Partly

Reviewer Expertise:

Entrepreneurship-Multiculturalism-Organisational Behaviour

I confirm that I have read this submission and believe that I have an appropriate level of expertise to confirm that it is of an acceptable scientific standard, however I have significant reservations, as outlined above.

# Response

**Published:** 2026-02-04

**Authors:** Marisleidy Alba

We sincerely appreciate the reviewer’s valuable and constructive feedback. In response, we have undertaken targeted revisions to enhance the theoretical clarity, methodological transparency, and practical relevance of the manuscript. We outline the implemented changes below and address each of the reviewer’s comments. All revisions are highlighted in green in the revised manuscript.

**Reviewer comment:**

 Further development of the theoretical framework is required, as well as greater clarity regarding the importance, purpose, and contributions of the study.

**Response:**

 These aspects were explicitly strengthened by clearly stating the study’s purpose, identifying the gap in the Latin American transnational literature, and specifying the study’s theoretical and practical contributions. In addition, the Theoretical Background was revised to include an integrative opening statement, an explicit description of the relationships among the variables supported by relevant empirical literature from the last 15 years, and a final synthesis linking the conceptual framework to the empirical analyses.

**Reviewer comment:**

 The abstract should indicate the purpose, methods, findings, and significance of the study.

**Response:**

 The Abstract was revised to explicitly state the study’s purpose, methodological approach, key findings (distinguishing entrepreneurial intention, discovery, and exploitation phases), and its practical relevance for curriculum design and university entrepreneurship ecosystems.

**Reviewer comment:**

 More detailed information is required regarding the sample and the participating universities.

**Response:**

 The Methods/ Participants section was expanded to clarify the sampling strategy, justify the use of ACBSP-accredited universities as a criterion for institutional comparability, describe the sample distribution by country, explain the multinational recruitment process, and outline the ethical reasons for not disclosing the names of the participating institutions.

**Reviewer comment:**

 The use of SPSS/AMOS and mediation or moderation analyses is suggested.

**Response:**

 We appreciate this suggestion. The study was intentionally designed as an exploratory and cross-national comparative analysis rather than a confirmatory model. In line with the ordinal nature of the data and the descriptive–explanatory objective, non-parametric analyses, correlational analyses, regression models, and visual analytics using raincloud plots were employed. Mediation and moderation analyses are identified as a direction for future confirmatory research.

**Reviewer comment:**

 Separating the Results and Discussion sections and strengthening theoretical, practical, and cultural contributions is recommended.

**Response:**

 This recommendation was implemented. The manuscript now presents separate Results and Discussion sections, and the theoretical, practical, and cultural implications were strengthened, including recommendations for university management and the design of context-sensitive institutional policies.

# Reviewer report: Reviewer response for version 1

**DOI:** 10.5256/f1000research.186207.r416191

**Published:** 2025-10-31

**Authors:** Alejandro Valencia-Arias

**Affiliations:** 1 Universidad Señor de Sipán, Chiclayo, Peru

It is recommended to include a broader theoretical background section that contextualizes the study within the Latin American literature on university entrepreneurial behavior. This would highlight the gaps addressed by the research and strengthen its theoretical contribution.

 The sample size and selection should be explained in greater detail, including the justification for the number of participants per country, the voluntary sampling criterion, and potential limitations of representativeness. Including a statistical power analysis or a theoretical reference would strengthen methodological validity.

 It is suggested to clearly separate the Results and Discussion sections, as empirical descriptions and theoretical interpretations are currently intertwined, making the analysis difficult to read and understand. A structural division would provide greater rigor and clarity of argument.

 The Discussion section should delve into the added value of the study, explaining its practical implications for universities in the region and comparing the results with relevant international research. This will highlight the unique contribution of the work.

 It is recommended to add a section on limitations and future lines of research, addressing aspects such as the cross-sectional design, the use of self-reports, and cultural differences between countries. This section would enhance transparency and provide guidance for future studies.

 The introduction could include explicit research questions or hypotheses, which better articulate the objectives with the methodology and results. This would increase the internal coherence of the manuscript and facilitate the assessment of theoretical validity.

 Although the use of raincloud plots is a visual success, it would be advisable to complement them with descriptive tables (means, standard deviations, and effect sizes) to allow for more precise interpretation and possible replication of the study.

Is the work clearly and accurately presented and does it cite the current literature?

Partly

If applicable, is the statistical analysis and its interpretation appropriate?

Yes

Are all the source data underlying the results available to ensure full reproducibility?

Yes

Is the study design appropriate and is the work technically sound?

Partly

Are the conclusions drawn adequately supported by the results?

Yes

Are sufficient details of methods and analysis provided to allow replication by others?

Partly

Reviewer Expertise:

entrepreneurial intention

I confirm that I have read this submission and believe that I have an appropriate level of expertise to confirm that it is of an acceptable scientific standard, however I have significant reservations, as outlined above.

# Response

**Published:** 2025-11-20

**Authors:** Marisleidy Alba

Dear Reviewer,

 We sincerely thank you for the time and effort dedicated to reviewing our manuscript. It is an honor to have our work read and evaluated by a scholar of your expertise. We have carefully considered all your comments and suggestions, and below we detail the revisions made in response to each of them.

***Comment: **It is recommended to include a broader theoretical background section that contextualizes the study within the Latin American literature on university entrepreneurial behavior. This would highlight the gaps addressed by the research and strengthen its theoretical contribution.*

**Response:** Thank you very much for your comment. In response to the reviewer’s recommendation, an expanded section on theoretical background has been incorporated to contextualize the study within the Latin American literature on University Entrepreneurial Behavior (UEB).

 This section synthesizes the main theories and quantitative findings developed in the region over the past 15 years, highlighting the predominance of the Theory of Planned Behavior (Ajzen, 1991) and the incorporation of competency-based approaches (Fernández-Pérez et al., 2019). It also analyzes cross-national comparative studies that integrate psychological, cognitive, and contextual variables in countries such as Mexico, Colombia, Chile, Peru, and Ecuador.

 Furthermore, it identifies existing gaps in the regional literature, such as the scarcity of longitudinal studies, the limited integration among psychological, cognitive, and contextual factors, the unequal geographical coverage, and the weak articulation between micro-level (individual cognition) and macro-level (institutional context) analyses.

 The section argues for the theoretical contribution of the study by proposing a model that integrates these factors within a causal and comparative framework for Latin America, thereby strengthening the understanding of university entrepreneurial behavior from a regional and updated perspective.

***Comment: **The sample size and selection should be explained in greater detail, including the justification for the number of participants per country, the voluntary sampling criterion, and potential limitations of representativeness. Including a statistical power analysis or a theoretical reference would strengthen methodological validity*.

**Response:** We appreciate the reviewer’s comment regarding the sample size and selection. The study employed a voluntary, purposive sampling strategy, coordinated with accredited universities in five Latin American countries, to ensure diversity among participants in terms of gender, academic level, and institutional background.

 The achieved sample sizes per country (Mexico = 168, Peru = 100, Ecuador = 147, Paraguay = 84, Colombia = 41; total N = 541) exceed the minimum thresholds recommended for cross-cultural comparative analyses (Field, 2013). To verify empirical adequacy, a post hoc power analysis was conducted for an omnibus comparison equivalent to the Kruskal–Wallis test (five groups, α = .05, medium effect f = 0.25 ≈ η² = 0.06; Cohen, 1988). The resulting statistical power was 0.97, indicating that the total sample was more than sufficient to detect effects of medium magnitude. Consistently, the descriptive results in Appendix A show low standard errors (SE ≤ 0.14) and small to moderate effect sizes (ω² and ε²), supporting the precision and stability of the estimates.

 Although voluntary sampling limits strict representativeness, diversity was maximized by recruiting from multiple ACBSP-accredited universities in each country. This approach aligns with methodological standards in cross-national research on entrepreneurial education (Fayolle & Gailly, 2015; López et al., 2021).

 Cohen, J. (1988). Statistical power analysis for the behavioral sciences (2nd ed.). Lawrence Erlbaum Associates.

 Fayolle, A., & Gailly, B. (2015). The impact of entrepreneurship education on entrepreneurial attitudes and intention: Hysteresis and persistence. Journal of Small Business Management, 53(1), 75–93. https://doi.org/10.1111/jsbm.12065

 Field, A. (2013). Discovering statistics using IBM SPSS statistics (4th ed.). SAGE Publications.

 López, F., Contreras, F., & Barbosa, D. (2021). Entrepreneurial intention in higher education: The role of personality traits and support mechanisms. International Journal of Management Education, 19(3), 100569. https://doi.org/10.1016/j.ijme.2021.100569

***Comment: **It is suggested to clearly separate the Results and Discussion sections, as empirical descriptions and theoretical interpretations are currently intertwined, making the analysis difficult to read and understand. A structural division would provide greater rigor and clarity of argument.*

**Response:** Thank you very much for the observation. The results have been separated from the discussion, which has now been developed into a dedicated Discussion section.

***Comment: **The Discussion section should delve into the added value of the study, explaining its practical implications for universities in the region and comparing the results with relevant international research. This will highlight the unique contribution of the work.*

**Response:** Thank you very much. A Discussion section has been incorporated, where the results are compared with relevant international studies. In this discussion, we show that by empirically linking attitudinal, cognitive, and contextual dimensions, our study advances toward a unified model capable of explaining how education and ecosystem experiences jointly sustain entrepreneurial behavior in university contexts.

***Comment: **It is recommended to add a section on limitations and future lines of research, addressing aspects such as the cross-sectional design, the use of self-reports, and cultural differences between countries. This section would enhance transparency and provide guidance for future studies.*

**Response:** The Limitations and Future Research Directions sections have been incorporated.

***Comment: **The introduction could include explicit research questions or hypotheses, which better articulate the objectives with the methodology and results. This would increase the internal coherence of the manuscript and facilitate the assessment of theoretical validity.*

**Response:** Thank you very much for the recommendation. The following research questions have been included:

 (1) What psychological, cognitive, and contextual factors explain entrepreneurial intention and action among business students from ACBSP-accredited Latin American universities?

 (2) How do cross-country differences reflect the influence of institutional and cultural contexts on students’ entrepreneurial profiles?

 (3) What implications do these findings have for curricular design and for strengthening university entrepreneurship ecosystems in the region?

***Comment: **Although the use of raincloud plots is a visual success, it would be advisable to complement them with descriptive tables (means, standard deviations, and effect sizes) to allow for more precise interpretation and possible replication of the study.*

**Response: **We appreciate the reviewer’s suggestion. In response, descriptive statistics (including means, standard deviations, standard errors, and effect sizes ω² and ε² for each variable and country) are presented in Appendix A. These tables complement the raincloud plots by providing numerical values that support precise interpretation and potential replication of the study.
